# 3D high-density microelectrode array with optical stimulation and drug delivery for investigating neural circuit dynamics

**DOI:** 10.1038/s41467-020-20763-3

**Published:** 2021-01-21

**Authors:** Hyogeun Shin, Sohyeon Jeong, Ju-Hyun Lee, Woong Sun, Nakwon Choi, Il-Joo Cho

**Affiliations:** 1 Center for BioMicrosystems, Brain Science Institute, Korea Institute of Science and Technology (KIST), Seoul, Republic of Korea; 2 Division of Bio-Medical Science and Technology, KIST School, Korea University of Science and Technology (UST), Seoul, Republic of Korea; 3 Department of Anatomy, Korea University College of Medicine, Seoul, Republic of Korea; 4 KU-KIST Graduate School of Converging Science and Technology, Korea University Seoul, Republic of Korea; 5 School of Electrical and Electronics Engineering, Yonsei University, Seoul, Republic of Korea; 6 Yonsei-KIST Convergence Research Institute, Yonsei University, Seoul, Republic of Korea

**Keywords:** Extracellular recording, Optogenetics, Neural circuits, Biomedical engineering

## Abstract

Investigation of neural circuit dynamics is crucial for deciphering the functional connections among regions of the brain and understanding the mechanism of brain dysfunction. Despite the advancements of neural circuit models in vitro, technologies for both precisely monitoring and modulating neural activities within three-dimensional (3D) neural circuit models have yet to be developed. Specifically, no existing 3D microelectrode arrays (MEAs) have integrated capabilities to stimulate surrounding neurons and to monitor the temporal evolution of the formation of a neural network in real time. Herein, we present a 3D high-density multifunctional MEA with optical stimulation and drug delivery for investigating neural circuit dynamics within engineered 3D neural tissues. We demonstrate precise measurements of synaptic latencies in 3D neural networks. We expect our 3D multifunctional MEA to open up opportunities for studies of neural circuits through precise, in vitro investigations of neural circuit dynamics with 3D brain models.

## Abstract (web summary)

Currently technologies for monitoring and controlling neural activities in 3D models are lacking. Here the authors report a 3D high-density multielectrode array, with optical stimulation and drug delivery, to investigate neural circuit dynamics in engineered 3D neural tissues.

## Introduction

Neural circuit dynamics is known as spatiotemporally varying activity patterns of synaptically-wired neurons that become active or silent. The investigation of neural circuit dynamics is essential for deciphering the functional connectivities among the regions of the brain for identifying the mechanisms of circuit dysfunctions related to brain diseases. While microphysiological systems (MPS; tissues/organs-on-chips) have emerged as increasingly promising tools in vitro for augmenting drug developments and for elaborating physiological and pathological states of the body^1^, such efforts for the brain have focused on reconstructions of neural networks or circuits on chips. The needs of these models in vitro continue growing because the models become complementary to animal experiments and can accomplish what in vivo tests cannot.

Recently, the developments of in vitro platforms have provided controllable environments for measuring inter-neuronal dynamics^2^. For example, the comparisons of neural dynamics between healthy and diseased model cells via two-dimensional (2D) cultures demonstrated the potential mechanisms associated with brain disorder-induced circuit dysfunctions^3–5^. However, 2D cell cultures, which are still used extensively, inherently cannot recapitulate the structure and functions of three-dimensional (3D) living tissues^6^. Especially for brain or neural tissues, a surge of interest in 3D cultures has occurred with the hope of developing both physiological and pathological models in vitro with the utilization of brain organoids, microphysiological systems (i.e., brain-on-chips), or 3D-printed, engineered tissues^7–11^. Specifically, the assembly of silk-based modular scaffolds seeded with cortical neurons allowed the building of multi-layered, 3D cortical tissue^12^. In addition, the directional alignment of the collagen microfibrils enabled the reconstruction of a functional hippocampal neural circuit in vitro at a 3D tissue scale^13^.

Despite the emerging advancements of engineered 3D neural circuit models, technologies for both precisely monitoring and modulating neural activities within the neural circuit models in vitro have not been developed yet. Calcium imaging or planar extracellular electrophysiology with a 2D microelectrode array (MEA), which are the tools that are commonly used in 2D cultures of neurons in vitro, remain the mainstream methodologies for monitoring neural activities in 3D in vitro models^14–20^. A significant disadvantage of these measurement techniques is the difficulty of analyzing the neuronal connections and the dynamics of the neural network in a 3D microenvironment. Similarly, the investigation of neural circuits in vivo remains limited due to the nature of 3D connectivity^21^.

As an excellent alternative, 3D MEAs have provided an opportunity to study neural networks in 3D brain models in vitro^22,23^. However, the 3D MEAs that have been reported to date have limitations in monitoring the neural circuit dynamics due to both low density^23^ and the randomly arranged^22^ recording sites. The previous 3D MEAs were also only capable of stimulating the surrounding neurons electrically; thus, stimulating specific cell types has been challenging^24,25^. However, the MEA with localized optical stimulation and drug delivery capabilities would help map functional connectivity in neural circuits in vitro by cell-type-specific stimulation and neurochemical modulation^26^. In addition, in 3D MEAs, the compact system is required to monitor the growth phases of developing neural networks in a temporally-resolved manner, for instance, by daily recordings^27–30^. This feature has been an advantage of the analyses of developing neuronal connections on a 2D MEA^27–32^ that can be accommodated in an incubator. Therefore, an ideal 3D MEA that can be used to investigate the neural circuit dynamics in vitro must satisfy the following requirements: spatial coverage across the total volume of an engineered 3D in vitro model, design flexibility according to types and sizes of 3D in vitro models (e.g., engineered neural tissues, organoids), high spatial resolution to analyze the functional connectivity among neurons in 3D in vitro models, localized optical and chemical stimulation capabilities for accurate modulations, and compact integration for temporally-resolved measurements in an incubator.

**Fig. 1:**
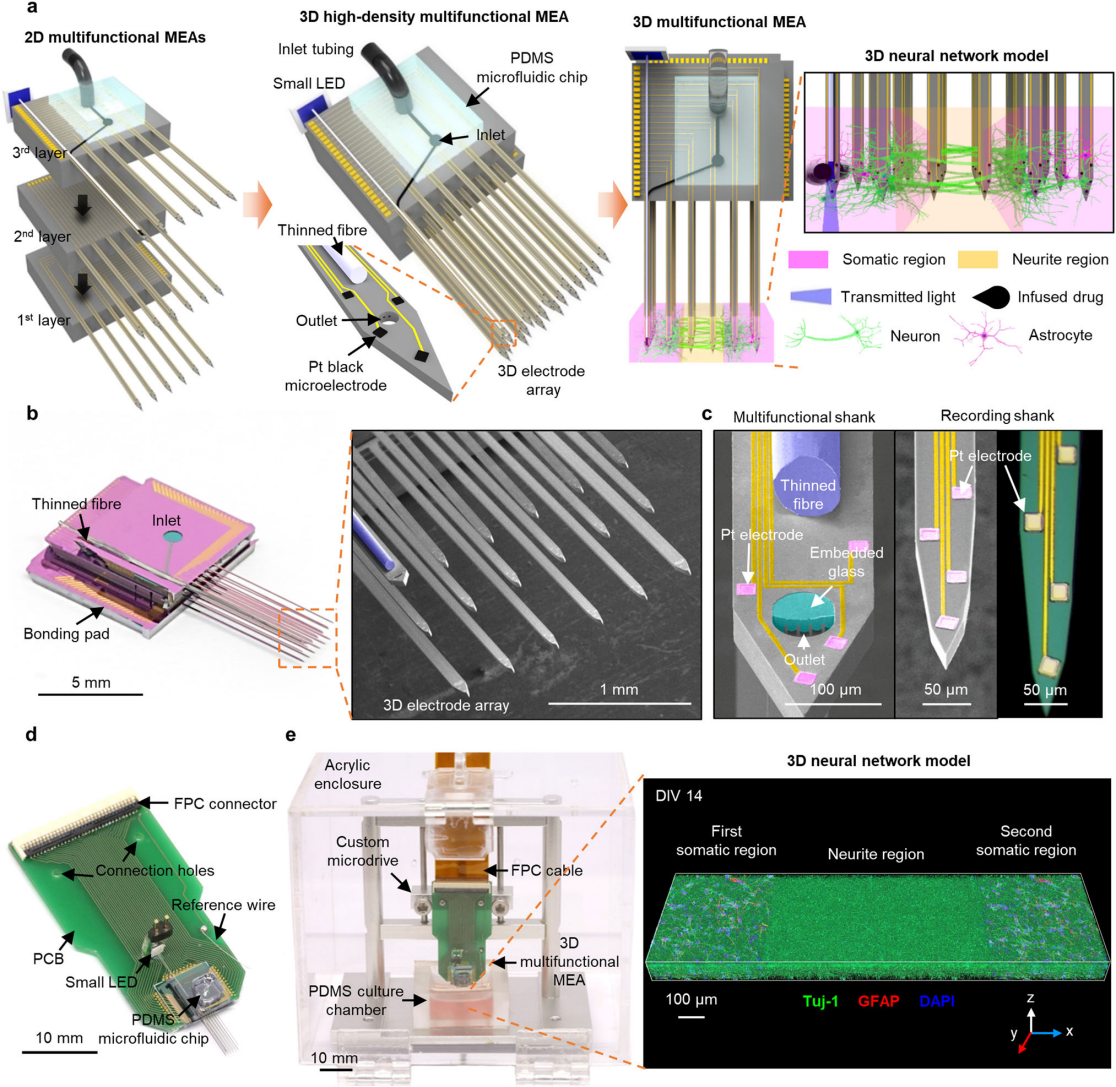
3D high-density multifunctional microelectrode array (MEA) system. **a** Schematic illustrations showing three 2D multifunctional MEAs before stacking and bonding (left), assembled 3D high-density multifunctional MEA with a PDMS fluidic interface and the multifunctional shank for optical and chemical stimulations (middle), and the application to 3D neural network model in vitro compartmentalized with two somatic and neurite regions (right). **b** Photograph of the 3D multifunctional MEA (left; scale bar, 5 mm) and scanning electron microscopy (SEM) image of the 3D electrode array (right; scale bar, 1 mm). **c** SEM image of the multifunctional shank with thinned optical fiber (blue), embedded glass (green) and outlet of microchannels underneath the embedded glass layer (left; scale bar, 100 μm), and SEM and optical images of the recording shanks with platinum (Pt) electrodes (right; scale bar, 50 μm). **d** Photograph of packaged 3D multifunctional MEA integrated with small light-emitting diode (LED) and flexible printed circuit (FPC) connector on a printed circuit board (PCB). (Scale bar, 10 mm). **e** Photograph of the 3D multifunctional MEA system with a custom microdrive and a PDMS 3D culture chamber in an acrylic enclosure (left; scale bar, 10 mm), and 3D rendered confocal fluorescence image of the compartmentalized two-group neural network at day 14 in vitro (DIV) showing neurites (green; Tuj-1), astrocytes (red; GFAP), and nuclei (blue; DAPI). Scale bar, 100 μm. Fabrication and packaging of the MEA system were independently repeated at least ten times with similar results to ensure reproducibility, and the representative images are shown in the figure.

To address the challenges listed above, we present a 3D multifunctional MEA system integrated with a 3D high-density microelectrode array, a thin optical fiber coupled with a small light-emitting diode (LED) and microfluidic channels, both of which are embedded in a shank for precise modulation of neural networks, and a miniaturized incubating and recording system for daily recordings of the developing neural networks (Fig. 1). The high-density array of electrodes integrated on the multi-shank structure of the 3D MEA allows the dynamics of the neural network to be measured from a compartmentalized neural tissue. The thin optical fiber and microfluidic channels integrated on our 3D MEA enable precise investigation of the functional connectivity between different neuronal groups through locally optical stimulation and drug delivery. Due to its miniaturized packaging, the incubating and recording system provides a suitable environment for the investigation of temporal evolutions in the dynamics of developing neural networks. Consequently, our 3D multifunctional MEA offers pivotal functions for the precise analysis of 3D brain models in vitro. In order to demonstrate the capabilities of our 3D multifunctional MEA, we analyze the temporal evolutions in neural circuit dynamics over two weeks within a compartmentalized 3D neural tissue where the functional connectivity formed between two different populations of cortical neurons. Also, we measure the synaptic latencies and transmission velocities of neural networks within a compartmentalized 3D neural tissue, which is enabled by both a high density of electrodes and precise stimulating modulations with localized optical stimulation and drug delivery capabilities of our multifunctional 3D MEA. Furthermore, we measure the synaptic latency and transmission velocity from an in vitro 3D culture of neurons. We expect that this 3D multifunctional MEA can open up various opportunities for studies of both neural circuits and neurological disorders through precise investigations of neural circuit dynamics with 3D brain models in vitro.

## Results

### Design and fabrication of the 3D multifunctional MEA

Our 3D multifunctional MEA consists of a three-by-six array of shanks, i.e., one multifunctional shank and 17 recording shanks. We integrated 63 recording microelectrodes evenly throughout the 18 shanks and embedded both a thinned optical fiber (~60 μm in diameter) and five parallel microfluidic channels in the multifunctional shank (Fig. 1a and 1b). The integration of all the electrodes, the optical fiber, and the microfluidic channels allowed measurements of neural activities across the whole region within an engineered 3D neural tissue, as well as measurements of the local modulations of the neural networks at a specific site via optical and chemical stimulations (Fig. 1a). Each shank was 6 mm long, which was enough to provide space between the 3D neural tissue and the printed circuit board (PCB) of a packaged device. Our intent was to prevent accidental short circuits of the PCB immersed in the culture medium, as well as potential contamination of the medium. To maintain the 3D neural tissue’s structural fidelity with 3D MEA implanted, we aimed to minimize the probe’s dimensions. The multifunctional shank’s width was 145 μm, the recording shank’s width was 63 μm, and the shanks’ thicknesses was 40 μm. The proportion of volume occupied by our 3D MEA was only 2.63% of the total volume of the 3D neural tissue, which suggests that the pre-inserted 3D MEA would affect 3D neural networks’ formation in a nearly negligible manner. When viewed from the bottom, the shanks of our 3D MEA formed a matrix with three rows and six columns (Figs. 1a and 1b). The spacings of the rows and columns were 500 and 360 μm, respectively. We integrated either three or four recording microelectrodes (20 × 20 μm^2^) on the tip of each shank, and the distance between two adjacent electrodes was 85 μm. We designed the 3D MEA to cover the dynamics of neural network within a monolithic, yet compartmentalized, 3D neural tissue construct (1.85 × 1 × 0.3 mm^3^) comprised of a neurite region between two somatic (Fig. 1a and 1b). Notably, the volumetric coverage to which the microelectrodes corresponded was approximately 114 sites⋅mm^-3^. The density of the recording sites was ~33 sites⋅mm^−3^, which was significantly higher than that of a 3D MEA that was reported recently^23^.

We devised a 3D multifunctional MEA with three layers of 2D MEAs that were fabricated separately using our previous microengineering processes^26,33,34^ (Supplementary Fig. 1). Specifically, we assembled a 3D configuration by bonding the three 2D MEAs consecutively with different body sizes (Fig. 1a and Supplementary Fig. 2). A highlight of fabricating the multifunctional shank of the 2D MEA was when the microfluidic channels for the delivery of the chemicals were embedded directly in a thin shank^34^. We placed an outlet that had a width and a length of 30 μm and 12 μm, respectively, at the tip of the multifunctional shank where each of the three 20 μm wide, 12 μm high microchannels ended (Fig. 1c). Note that our implementation readily allowed the highly flexible configuration of the microelectrodes by precise adjustment of the positions of the shank on the 2D MEA arrays, as well as in a 3D setting, e.g., a setting with inter-body spacers in between^33,35^ (Supplementary Fig. 2). Overall, our design and fabrication of the 3D multifunctional MEA provides the capability of local, multimodal manipulation of neural networks during millimeter-scale monitoring of neural activities across the entire domain of 3D neural tissues in vitro.

### Packaging and characterization of the 3D multifunctional MEA

Figure 1d shows the 3D multifunctional MEA that we packaged for the in vitro experiments, which was done by bonding on a custom-designed PCB for electrical connections to an external recording system and assembly with a custom microdrive. Then, we bonded a polydimethylsiloxane (PDMS) microfluidic chip onto the 3D MEA as a fluidic interface for the delivery of drugs. Then, we bonded a small LED to the end of the fiber. The LED provided a simple operating environment in which only two thin electrical wires were required instead of any external light sources, such as a laser. After the packaging, we enhanced the neural recording capability by increasing the effective surface areas of the platinum (Pt) electrodes by electrodepositing Pt-black^36^ on them (Supplementary Fig. 3a).

We evaluated three crucial functions of our 3D multifunctional MEA before analyzing the dynamics of the neural network. First, we measured the electrical impedance of all 63 of the microelectrodes. We confirmed that the average electrical impedance after the electrodeposition of the Pt-black at 1 kHz (0.015 ± 0.004 MΩ) became two orders of magnitude lower than that of bare Pt (1.761 ± 0.346 MΩ) (Supplementary Fig. 3b). This significantly reduced the impedance, i.e., ~44 times lower than iridium (Ir) electrodes that were the same size^26^, which was much more advantageous for the extracellular measurements of neural activities, acquiring higher signal-to-noise ratios. To evaluate the Pt-black electrodes’ long-term stability, we measured the electrical impedance of Pt-black electrodes submerged in 1× phosphate-buffered saline (PBS) in an incubator at 37 °C. We confirmed that the impedance remained nearly constant from day 1 (0.017 ± 0.004 MΩ) to 14 (0.018 ± 0.004 MΩ) without degradation (Supplementary Fig. 3c).

Next, we measured the flow rates through the microfluidic channels for three different conditions, i.e., with the 3D MEA exposed to air, with the 3D MEA inserted in cell-free collagen, and with the 3D MEA inserted in cell-seeded collagen. We applied pressure between 50 and 200 kPa from an inlet of the PDMS chip. We chose 0.25% [w/v] of collagen type I because it has used extensively as a scaffold type and concentration for engineered tissue constructs^37^, including neural tissues^13^. For the cell-laden collagen, we seeded primary rat cortical neurons (E18) at a density of 4 × 10^7^ cells⋅mL^−1^, which was identical to the in vitro experimental conditions used throughout this study. At a pressure of 100kPa, the flow rates at these conditions were 0.557 ± 0.007 μL⋅min^−1^ (in air), 0.542 ± 0.011 μL⋅min^−1^ (in cell-free collagen), and 0.539 ± 0.015 μL⋅min^−1^ (in neuron-seeded collagen), all of which were statistically insignificant and similar to the calculation based on hydraulic resistance^26^, i.e., 0.568 μL⋅min^−1^ (Supplementary Fig. 3d). These data verify that we were able to control the infused volume precisely based on the predictive calculation.

Last, to predict the volumetric coverage of optical stimulation, we measured output optical power at the tip of the fiber and then simulated the distribution of transmitted light. First, referring to the LED datasheet, we calculated the luminous efficiency of the LED as 5.15% (Supplementary Fig. 3e and the detailed calculation in Supplementary Note. 1). As a result, we confirmed that the LED’s output optical power was 51.5 mW when 1 W of input electrical power was supplied to the LED. Upon 51.5 mW from the LED, the output optical power from the fiber tip was 0.15 mW, which corresponded to a light coupling efficiency of 0.29% (Supplementary Fig. 3e and the detailed calculation in Supplementary Note. 1). The light coupling efficiency was 0.29%, lower than 1.42%, as we previously reported^26^, but the proposed probe was integrated with a non-coherent LED instead of a coherent laser as a light source. The LED’s integration provided a compact and straightforward configuration while the LED exhibited inherently lower optical power due to poor directionality. Also, the optical power measured from the fiber tip corresponded to an optical power density of 76 mW⋅mm^−2^, which surpassed the minimum optical intensity (1 mW⋅mm^−2^) to activate channelrhodopsin-2 (ChR-2)^38^. Based on the measured output optical power (0.15 mW) transmitted from the LED when the input electrical power was 1 W, corresponding to the optical power of 51.5 mW, we used the Monte Carlo simulation^39,40^ to profile the volume of transmitted light in collagen. (More detailed simulation method is provided in the “Methods “section) We confirmed that the irradiance at a distance of 220 μm from the end of the fiber was greater than 1 mW⋅mm^−2^, which is the threshold intensity for the activation of channelrhodopsin-2 (ChR2) (Supplementary Fig. 3f). In addition, by inspecting the distribution of light in the *x*–*y* plane, we confirmed that the volumetric coverage that was stimulated did not extend beyond the tip of the shank. These data indicate that our 3D MEA allowed optical stimulation locally at the desired site (Supplementary Fig. 3f).

### Assembly of the monitoring system and the 3D neural culture

To enable real-time recording of neural activities within engineered neural tissues, we devised a miniaturized cubicle in which we assembled the 3D multifunctional MEA (Fig. 1e). The miniaturized cubicle consisted of the following: a custom-designed microdrive with stainless steel to adjust and hold the vertical position of our 3D MEA, a PDMS culture chamber with a well (2.5 × 1.5 × 0.5 mm^3^) to confine the neuron-seeded collagen scaffold and to supply culture medium, and an acrylic enclosure (10 × 8 × 8 cm^3^) to minimize the undesired evaporation of the medium over culture periods and during measurements of the dynamics of the neural network.

The custom-designed microdrive consisted of a moving part and bottom plate assembled by inserting two screws into the connection holes and tightening them (Supplementary Fig. 4a). By turning a long screw in the center of the moving part of the microdrive, the vertical position of the 3D MEA could be adjusted readily and precisely (Supplementary Fig. 4b). The overall system’s size was small and, the configuration, including the 3D multifunctional MEA, was straightforward (Supplementary Figs. 5 and 6). In addition, because the MEMS-based batch process allows for fabricating 20–30 electrode arrays from a single wafer, our system’s scaling-up to perform multiple simultaneous experiments would be reasonably feasible.

For the fabrication of 3D neural network model in our culture chamber as a first step, we immobilized the PDMS culture chamber on a bottom plate of the custom microdrive with a thin layer of uncured PDMS as an interfacial adhesive (Supplementary Fig. 6a). After autoclaving the culture chamber attached to the microdrive, we coated polydopamine on the inner surfaces of the PDMS well to adhere to the collagen scaffold. Then, we used two small screws to assemble the 3D MEA on a moving part of the microdrive (Supplementary Fig. 6b).

We found that the timing of the 3D MEA loading into the collagen scaffold was the most critical variable. More specifically, the 3D MEA should be loaded before initiating the gelation of the collagen in order to allow the uniform distribution of the collagen microfibrils near the shanks (Supplementary Fig. 7a and Supplementary Movie 1). In contrast, it was difficult to penetrate the 3D MEA by the insertion of the 3D MEA into collagen after complete gelation because of the fibrous, viscoelastic properties of the collagen, and the result exacerbated the structural deformation (Supplementary Fig. 7b and Supplementary Movie 1).

We developed two types of models of a 3D neural network, i.e., a single-group neural network and a compartmentalized two-group neural network (Supplementary Fig. 8). We used the single-group model for the analysis of the connectivity among the small units (i.e., individual neurons), and we used the two-group model for the analysis of the connectivity among the larger units at the network level. The compartmentalized neural tissue was formed by first consecutively filling cell-free collagen into a central compartment and then adding neuron-seeded collagen into the two side compartments at intervals of 20 min. In other words, we loaded the neuron-seeded collagen into the side compartments after partial gelation of the cell-free collagen in the central compartment for 20 min. We utilized ~125 μm-thick polyester (PET) films to separate the compartments in the well. (See Supplementary Fig. 8 and the more detailed protocol in the “Methods” section.) We noted that removal of the PET sheets in less than 40 min (e.g., 30 min; 10 min after loading in the side compartments) caused bleed-through between the side and central zones.

In the miniaturized cubicle, we confirmed that the primary rat cortical neurons in collagen maintained high viability (Supplementary Fig. 9 and Supplementary Movie 2) and formed structural connectivities with the transiently maturing outgrowth of neurites over two weeks in both the single-group and two-group neural network models (Supplementary Figs. 10, 11 and Supplementary Movies 3, 4). Interestingly, we found small portions of GFAP-positive astrocytes in both types of neural tissues, although we isolated neuron-rich populations from embryonic brains (E18) (Supplementary Figs. 10, 11, and 12). We also note that our compartmentalization approach was successful based on the neuronal nuclei that remained in the two somatic regions, while neurites spread through the central neurite region (Supplementary Figs. 11, 12 and Supplementary Movie 4). We note that 0.25% [w/v] collagen allows for uniform neuronal density throughout the full thickness of 300 μm because *z*-stack imaging through 100 μm showed uniform seeding density in both current and our previous studies^13^. However, lower concentrations of collagen or Matrigel lead to seeded neurons’ sedimentation by gravity before the scaffolds’ gelation. Unfortunately, due to the high cell seeding density (i.e., 4 × 10^7^ cell⋅mL^−1^) used in this study, which was similar to the cell density in the rat’s brain^41^, the *z*-stack imaging was limited to 100 µm with conventional imaging methods such as a confocal microscope. Advanced 3D volumetric imaging techniques such as a tissue clearing approach could serve as an excellent solution to overcome this limitation.

### Dynamics of neural network in 3D neural culture

**Fig. 2:**
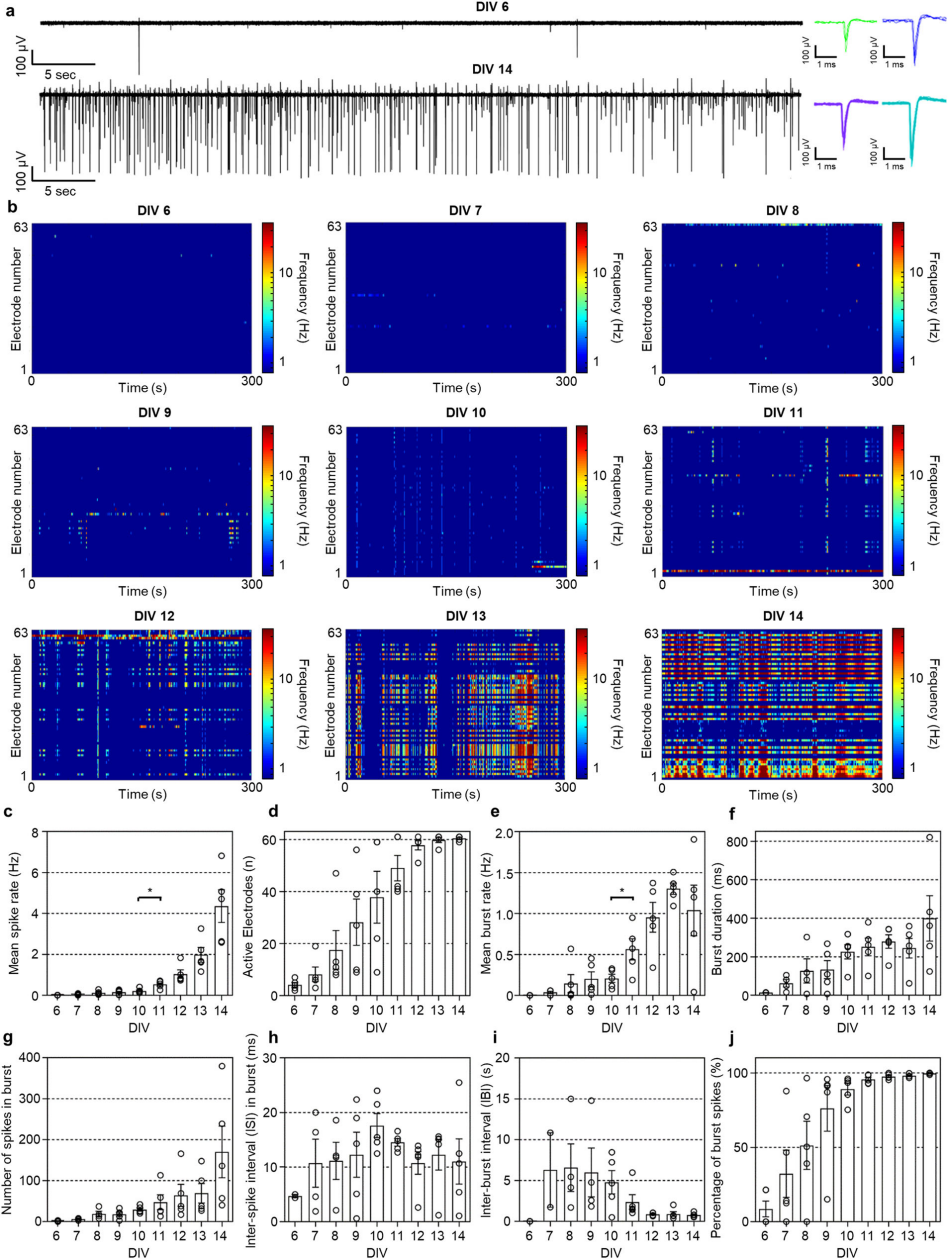
Temporal evolutions of spontaneous activities in the single-group 3D neural network model according to the formation of functional connections between neurons. **a** Representative spontaneous activities at days in vitro (DIV) 6 and 14: traces of field potential (left) and overlay spike plots (right). **b** Color-mapped raster plots showing spontaneous activities recorded from 63 electrodes of the 3D multifunctional MEA from DIV 6 to 14. **c**–**j** Bar graphs displaying temporal evolutions of spontaneous activities from DIV 6 to 14 (*n* = 5 independent samples for all data): mean spike rate (**c**; *P = 0.0102 between DIV 10 and 11), number of active electrodes (**d**), mean burst rate (**e**; *P = 0.032 between DIV 10 and 11), burst duration (**f**), number of spikes in burst (**g**), inter-spike interval (ISI) in burst activity (**h**), inter-burst interval (IBI) (**i**), percentage of burst spikes in total spikes (**j**). Data are presented as mean values +/− s.e.m. Statistical significance was tested with a two-tailed unpaired *t*-test. Source Data is available as a Source Data file for Fig. 2b-j.

To analyze the dynamics of neural networks according to the formation of functional connections between neurons in 3D, first, we measured the neural activities in the single-group model for up to 14 days in vitro (DIV). Spontaneous activities started showing up from DIV 6 on some electrodes, and both the neurons’ firing rates (Fig. 2a, color-mapped raster plots in Fig. 2b and mean spike rates in Fig. 2c) and the number of active electrodes (Fig. 2d) increased as we continued recording daily for 14 days. (See Supplementary Fig. 13.) These data are consistently similar to those from 2D and 3D in vitro neural models^27–31^, which reaffirmed that sufficient maturation, primarily including substantial neurite outgrowth and inter-neuronal synapse formation, is essential for the functional activity of neurons cultured in vitro^27,42–44^. Also, a thorough inspection of each electrode’s signals indicated that the neurons’ firing rates in 3D increased globally within the entire neural tissue (Supplementary Fig. 14).

In addition to the individual activities, our 3D high-density MEA made it possible to measure the frequency of the in vitro burst activity, i.e., the repeated high-frequency firing of neurons. Burst activity is an essential factor in neuronal communications both in vitro and in vivo^45,46^. While the neurons’ burst activity gradually increased until DIV 10, the overall frequency of the burst activity and the ratio of the burst activity within each neuron’s firing pattern increased after DIV 10 (Fig. 2e–2j). These results support the functional connections among neurons formed in 3D. Although, in general, the burst activity in vitro could be observed by increasing the density of cell seeding, our 3D MEA, with its densely integrated electrodes, dramatically increased the probability of capturing the burst activity, and it also enhanced the spatial resolution in precisely analyzing neural networks in vitro.

**Fig. 3:**
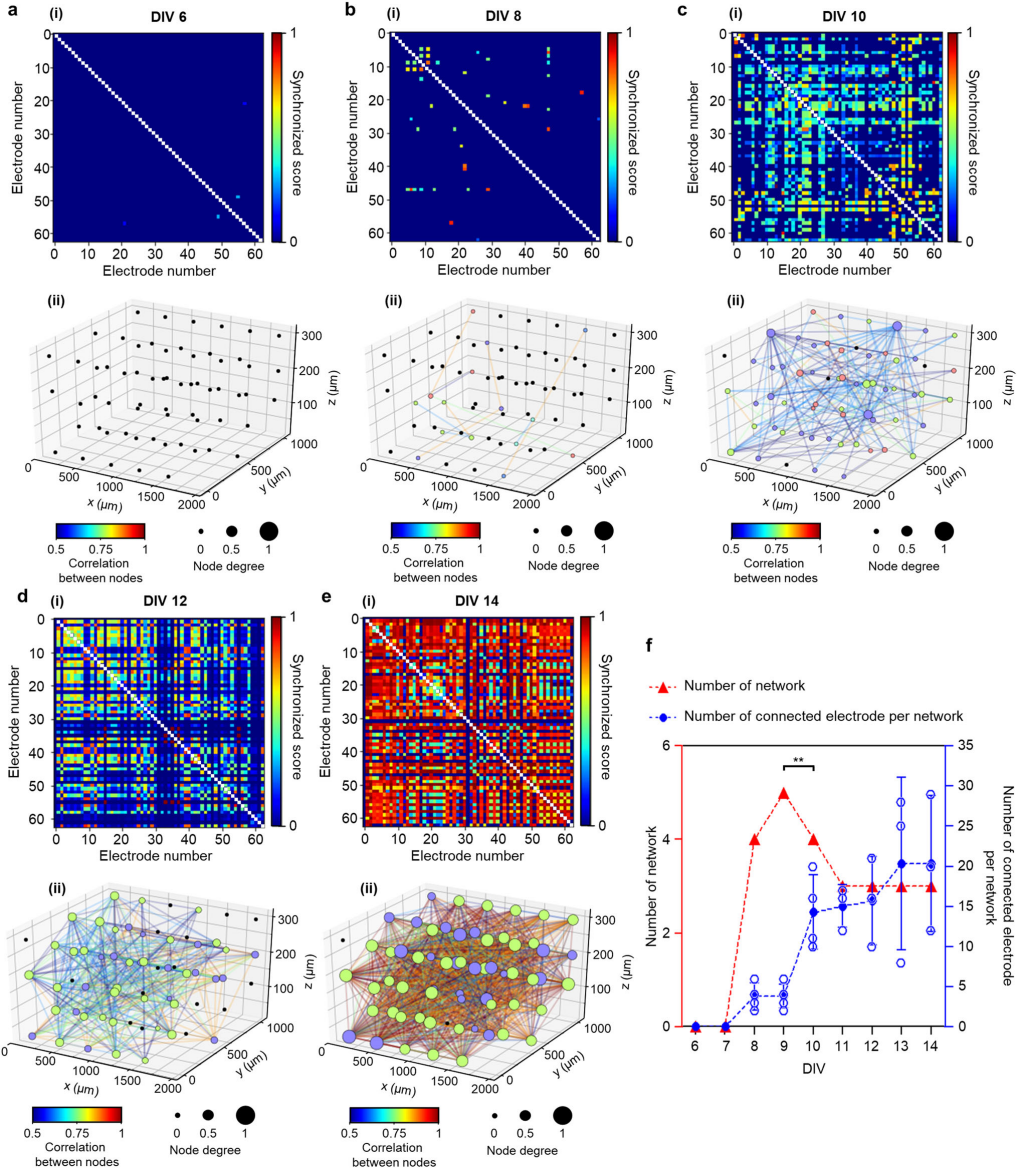
Analysis of neural network dynamics based on spontaneous activities in the single-group 3D neural network model. **a**–**e** Color-mapped cross-correlation matrices displaying synchronized scores between electrodes (**i**) and 3D network maps showing connectivities with node degrees, as well as correlations between nodes (**ii**), based on spontaneous activities at days in vitro (DIV) 6 (**a**), 8 (**b**), 10 (**c**), 12 (**d**), and 14 (**e**). The color of the node indicates the network index connected among electrodes; for example, the purple nodes at DIV 14 indicate that the nodes are in the same network. Node degree indicates the number of connected electrodes from each electrode; for example, the greatest value of 1 represents that the electrode is connected with all electrodes. The colors of the lines indicate the correlation between electrodes. **f** Plots showing the number of networks (red triangle and dotted line; **P = 0.002 between DIV 9 and 10) and the number of connected electrodes per network (blue circle and dotted line) from DIV 6 to 14. Data are presented as mean values +/− s.d. with individual data points (blue outlined circle; *n* = 3 networks for DIV 11, 12, and 14; *n* = 4 networks for DIV 8, 9, 10, and 13). Statistical significance was tested with a two-tailed unpaired *t*-test. Source Data is available as a Source Data file for Fig. 3a–f.

We also analyzed the synchronized activity that is known to occur in mature neural networks in vitro. We used the synchrony method, which assigns a score between zero (the lowest) and one (the highest), indicating the level of the synchronization to each pair of electrodes^32,47^. A high synchronization score suggests the formation of a functional neural network among the neurons around two electrodes. We confirmed that, as the culture period increased, the synchronization among active electrodes became more pronounced, and the number of synchronized electrodes also increased (Fig. 3a–e and Supplementary Fig. 15). These data indicated that the number of synapses among the neurons in the single-group neural network model in vitro increased tremendously after DIV 10. We also applied the Louvain algorithm^32,48^, a graphical network analysis, for 3D visualization of the functional neural network between neurons. We set each electrode as a node, synchronization between electrodes as weight, and merged them into possible networks when the synchronization score was greater than 0.5 between nodes. We found that a small number of networks started forming from DIV 8 (Fig. 3b). Intriguingly, the number of networks peaked at DIV 9, and this was followed by slight decreases later in vitro (Fig. 3f and Supplementary Fig. 15). These data indicated that short connections form increasingly between adjacent neurons over an early stage before DIV 10, and the short connections become relatively large networks that become mature by decreasing the number of networks while connections per network increased continuously (Fig. 3, Supplementary Fig. 15, and Supplementary Table 1).

### Dynamics of neural network with optical and chemical stimuli

**Fig. 4:**
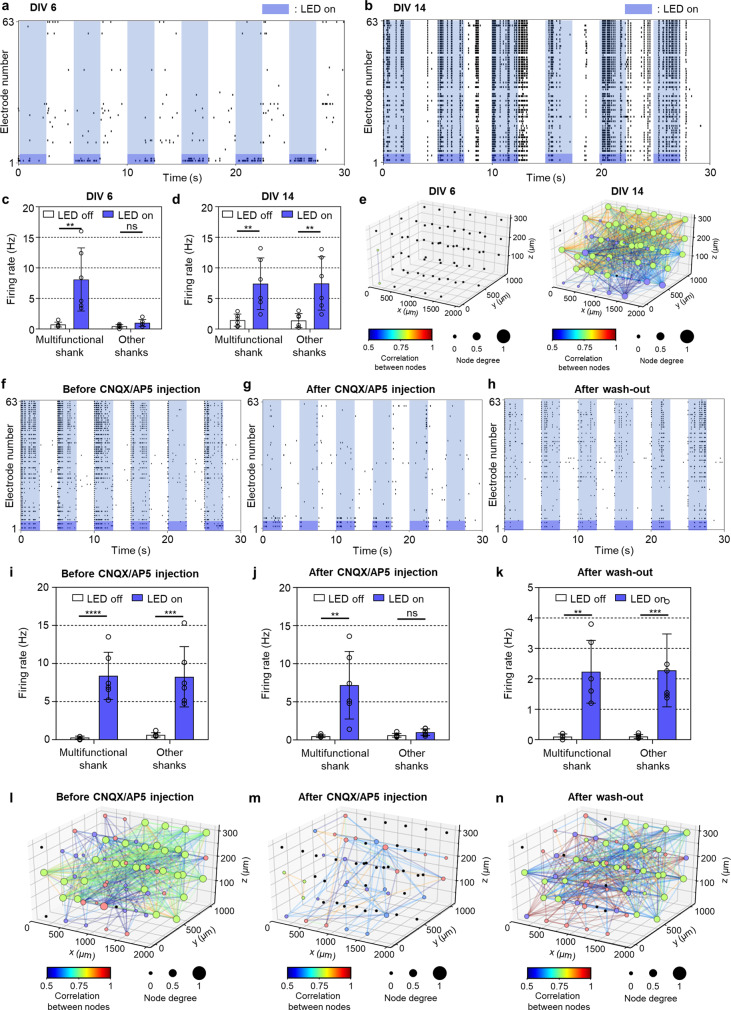
Analysis of synaptic connectivity before and after forming functional neural networks in the single-group 3D neural network model. **a**–**b** Raster plots showing neural activities recorded from 63 electrodes during local optical stimulations (0.2 Hz, 50% duty cycles) at days in vitro (DIV) 6 (**a**) and 14 (**b**). The light blue rectangle indicates the onset of light. The dark blue rectangle represents the electrodes on the light-transmitted multifunctional shank. **c**–**d** Firing rate of 3D cultured neurons near the multifunctional and other recording shanks during LED off (white)-cycle and on (blue)-cycle of the optical stimulations at DIV 6 (**c**; **P = 0.0058 near the multifunctional shank; ns *P* = 0.058 near the other recording shanks) and 14 (**d**; **P = 0.0072 near the multi-functional shank; **P = 0.0080 near the other recording shanks). **e** 3D network maps between electrodes based on neural activities by the optical stimulations at DIV 6 (left) and 14 (right). Node color, degree, and line color indicate network index connected among electrodes, the number of connected electrodes from each electrode, and the correlation between electrodes, respectively. **f**–**h** Raster plots showing neural activities recorded from 63 electrodes during local optical stimulation before (**f**) and after (**g**) CNQX/AP5 injection, and after wash-out CNQX/AP5 (**h**) at DIV 14. **i**–**k** Firing rate of 3D cultured neurons near the multifunctional and other recording shanks during LED off (white)-cycle and on (blue)-cycle of the optical stimulations before (**i**; ****P = 0.000079 near the multifunctional shank; ***P = 0.0008 near the other recording shanks) and after (**j**; **P = 0.0041 near the multifunctional shank; ns *P* = 0.0707 near the other recording shanks) CNQX/AP5 injection, and after wash-out CNQX/AP5 (**k**; **P = 0.0012 near the multifunctional shank; ***P = 0.0005 near the other recording shanks) at DIV 14. **l**–**n** 3D network maps between electrodes based on neural activities by the optical stimulations before (**l**) and after (**m**) CNQX/AP5 injection and after washing-out CNQX/AP5 (**n**) at DIV 14. Data are presented as mean values +/− s.d. with individual data points (white circle; *n* = 6 stimulation trials for all data). Statistical significance was tested with a two-tailed unpaired *t*-test. Source Data is available as a Source Data file for Fig. 4a–n.

For the precise investigation of the synaptic connectivity before and after the formation of the functional neural network in 3D, we utilized optogenetics as a verifying tool capable of cell type-specific stimulations^38^. After expressing a light-sensitive ion channel, i.e., channelrhodopsin-2 (ChR2), in the primary rat cortical neurons cultured within collagen by a viral infection with AAV-EF1α-ChR2-eGFP (Supplementary Fig. 16 and Supplementary Movie 5), We successfully measured activated neural signals from ChR2-neurons by light stimulation (0.2 Hz with a 50% duty cycle, 76 mW⋅mm^−2^ with a total power of 0.15 mW) despite the stimulation artifact by the photovoltaic effect during the transition between the light ON and OFF^49,50^ (Supplementary Fig. 17). After observing the activated neural signals by light stimulation, we stimulated the ChR2-neurons locally around the multifunctional shank while recording from all of the shanks of our 3D MEA. At DIV 6, only the neurons around the optical stimulation site correspondingly fired more (Fig. 4a, 4c, and Supplementary Fig. 18a). In contrast, when the optical stimulation occurred, the neurons at DIV 14 fired enormously throughout all of the neural tissue, as well as around the stimulation site (Fig. 4b, 4d, and Supplementary Fig. 18b). These results indicated that most of the neurons that had matured over two weeks in the functional network can be activated through synaptic connections (Fig. 4e and Supplementary Table 2) despite a slight decrease in spontaneous activity (i.e., neural activities during LED-off). This reduced activity is conceivably attributed to a potential effect on viability and neural activities by the ChR2-viral infection^51,52^, compared with the activities from the uninfected cell culture shown in Fig. 2.

To demonstrate the chemical activation of neurons by synaptic transmission combined with optogenetic modulation, at DIV 14, we infused synaptic blockers of excitatory synaptic transmission through the microfluidic channels in the multifunctional shank. Specifically, we injected 1 μL of CNQX (20 μM) and AP5 (50 μM) for 4 min at a flow rate of 0.25 μL⋅min^−1^ and waited 30 min to ensure that sufficient diffusion had occurred. The spontaneous neural activities are known to recover quickly upon exposure to CNQX/AP5^53^, but the synaptic transmissions are blocked until washed out^54^. Likewise, the mean firing rate during LED off remained similar before and after the CNQX/AP5 injection (Fig. 4i and 4j). Whereas neural activities (i.e., active electrodes and firing rate) were synchronized away from the stimulation site before the chemical silencing (Fig. 4f, 4i, and Supplementary Fig. 18c), when the light pulses were applied, the region of synchronized neural activities became limited predominantly to the region around the site of the optical stimulation (Fig. 4g, 4j, and Supplementary Fig. 18d). After washing out the synaptic blockers with fresh medium three times for 30 min followed by 1 h of stabilization, the neural activities re-synchronized throughout the 3D neural tissue (Fig. 4h, 4k, and Supplementary Fig. 18e). In addition, our network analysis indicated that the blockage of the excitatory synaptic receptors segregated more extensive networks (Fig. 4l) into several smaller networks (Fig. 4m and Supplementary Table 3); similarly, the synaptic networks recovered after the wash-out (Fig. 4n).

### Network dynamics between two compartmentalized neural groups

**Fig. 5:**
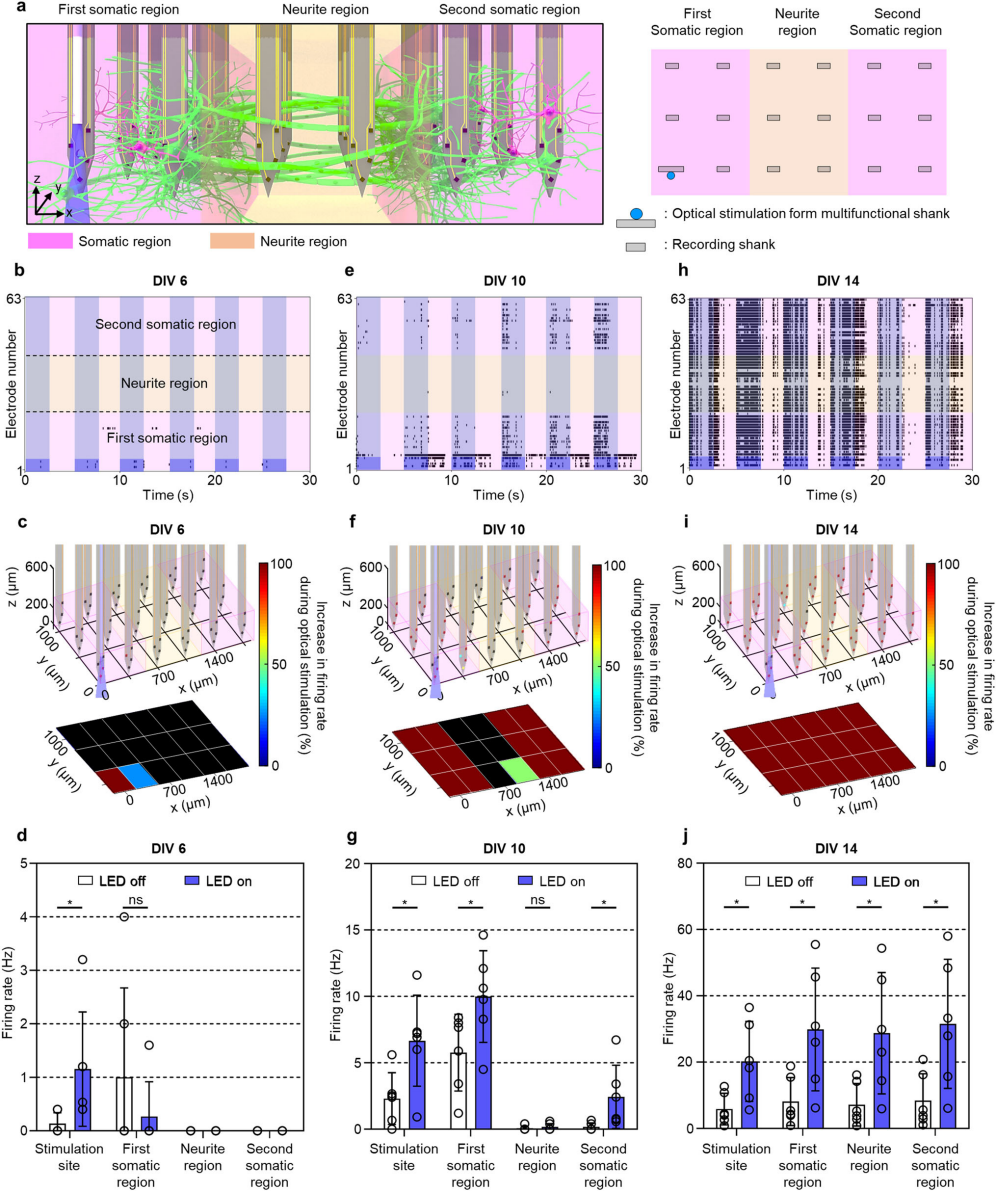
Analysis of functional connectivity between compartmentalized somatic regions in the two-group 3D neural network model. **a** Schematic illustrations of the 3D multifunctional MEA inserted in the compartmentalized 3D neural tissue consisting of two somatic regions (pink) and a neurite region (orange). Six shanks are positioned in each region, and the multifunctional shank is located near a corner of the first somatic region. The blue rectangle indicates the onset of light. **b**–**d** Changes in neural activities by locally optical stimulation at days in vitro (DIV 6) in the two-group 3D neural network model. **b** Raster plots displaying neural activities recorded from 63 electrodes of the 3D MEA within the compartmentalized regions during local optical stimulations (0.2 Hz, 50% duty cycles). The light blue rectangle indicates the onset of light. The dark blue rectangle represents the electrodes on the light-transmitted multifunctional shank. **c** 3D visualization of shank map in the compartmentalized neural network model (top) and *z*-averaged, color-mapped increase in firing rate during LED on-cycles, compared with that during LED off-cycles (bottom), corresponding to sub-regions near each shank as depicted in the top panel. Black indicates no signals recorded from the electrodes. **d** Firing rate of 3D cultured neurons at the stimulation site, in the first somatic, neurite, and the second somatic regions during LED off (white)-cycle and on (blue)-cycle of the optical stimulations (*P = 0.0443 at the stimulation site; ns *P* = 0.3409 in the first somatic region). **e**–**g** Changes in neural activities by locally optical stimulation at DIV 10 in the two-group 3D neural network model. **e** Raster plots; (**f**) 3D visualization of shank map and *z*-averaged, color-mapped increase in firing rate during LED on-cycles; (**g**) Firing rate of 3D cultured neurons at each region (*P = 0.0220 at the stimulation site; *P = 0.0444 in the first somatic region; ns *P* = 0.4506 in the neurite region; *P = 0.0436 in the second somatic region). **h**–**j** Changes in neural activities by locally optical stimulation at DIV 14 in the two-group 3D neural network model. **h** Raster plots; (**i**) 3D visualization of shank map and *z*-averaged, color-mapped increase in firing rate during LED on-cycles; (**j**) Firing rate of 3D cultured neurons at each region (*P = 0.0218 at the stimulation site; *P = 0.0229 in the first somatic region; *P = 0.0214 in the neurite region; *P = 0.0228 in the second somatic region). Data are presented as mean values + /− s.d. with individual data points (white circle; *n* = 6 stimulation trials for all data). Statistical significance was tested with a two-tailed unpaired *t*-test. Source Data is available as a Source Data file for Fig. 5b–j.

We examined the functional connectivity between two compartmentalized neural groups as an in vitro model for investigating neural circuit dynamics. The culture chamber for the two-group neural network model consisted of two side somatic regions separated by a central neurite region (Fig. 5a). Six shanks were located in each region to measure neural activities throughout the compartmentalized 3D neural tissue, while the shank with the stimulation capabilities was in the first somatic region (Fig. 5a). By immunostaining, visualization indicated that we had successfully reconstructed the soma-neurite-soma structure only with the dense outgrowth of neurites across the two somatic regions at DIV 14, starting from DIV 6 (Fig. 1e and Supplementary Fig. 11). Similar to the single-group model, the firing rate and the number of active electrodes increased substantially after DIV 10 (Supplementary Figs. 19 and 20). Also, our 3D MEA enabled the measurement of synchronized activity at DIV 9, which indirectly indicated that the functional connectivity between the two groups of cortical neurons had begun to form (Supplementary Fig. 19).

**Fig. 6:**
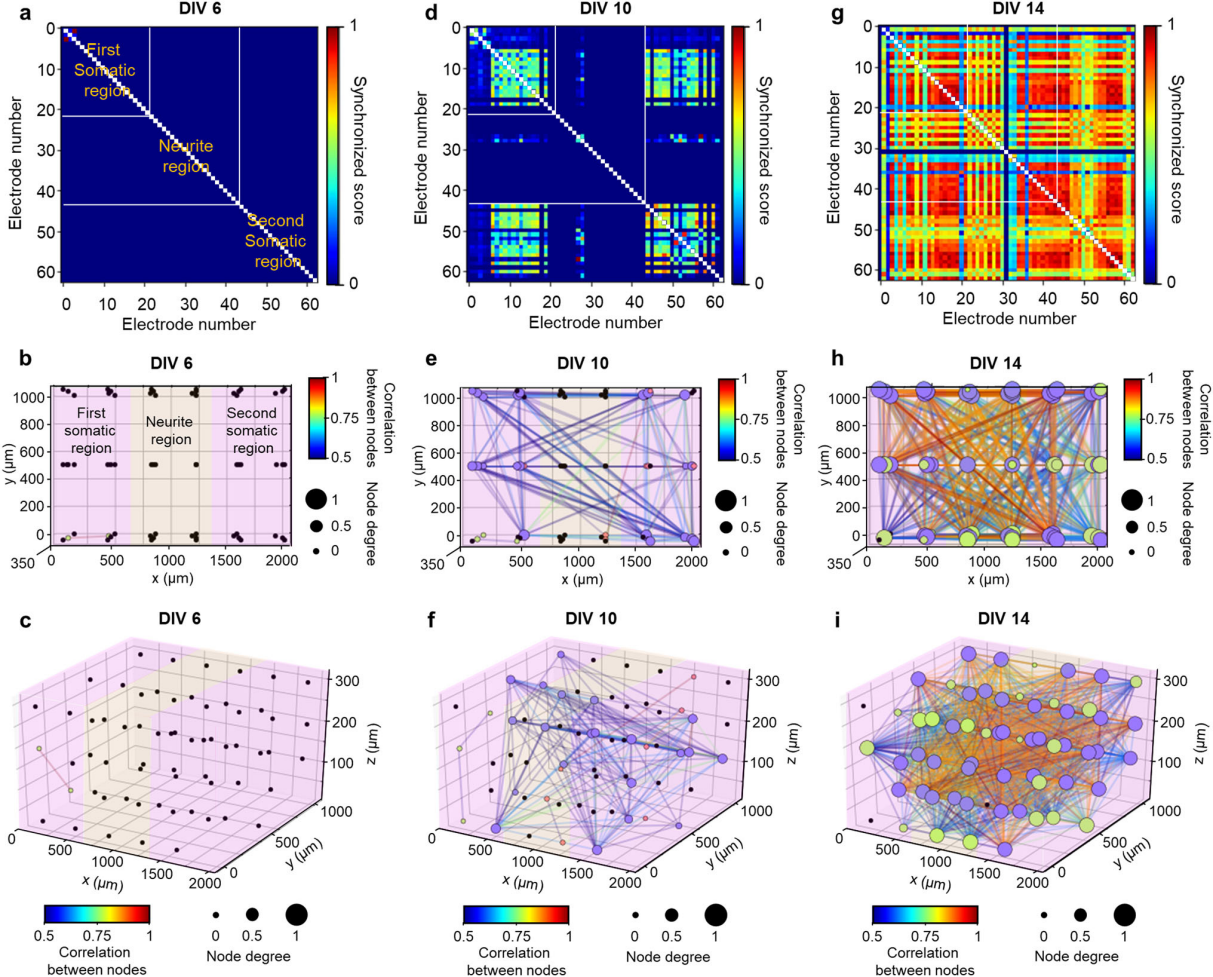
Temporal evolutions of neural network dynamics in the compartmentalized two-group 3D neural network model. Color-mapped cross-correlation matrices displaying synchronized scores between electrodes (**a**, **d**, and **g**), top-down view (*xy*-plane; **b**, **e**, and **h**) and 3D view (**c**, **f**, and **i**) of network maps showing connectivities with node degrees, as well as correlations between nodes, based on neural activities by optical stimulation at days in vitro (DIV) 6 (**a**–**c**), 10 (**d**–**f**), and 14 (**g**–**i**). Node color, degree, and line color indicate network index connected among electrodes, the number of connected electrodes from each electrode, and the correlation between electrodes, respectively. Source Data is available as a Source Data file for Fig. 6a–i.

To validate the formation of functional connectivity, we performed the spatially-resolved, local optical stimulation to the neurons expressing ChR2, followed by temporally-resolved daily measurements. At DIV 6, only neurons around the stimulation site were activated by the light pulses (Fig. 5b–d and Fig. 6a–c). Then, the optically-activated neurons gradually increased in the first somatic region between DIV 7 and 9 (Supplementary Fig. 21). At DIV 9, some neurons in the second somatic region became active soon after the optical stimulation of the first somatic region, which confirmed the formation of the functional connectivity between the two different neuronal populations (Supplementary Figs. 21 and 22). At DIV 10, we observed both significant increases in the firing rate in both regions by the optical stimulation and synchronized activities (Fig. 5e–g, Fig. 6d–f and Supplementary Figs. 22 and 23). Also, we observed a higher firing rate in the first than the second somatic region due to active burst activity recorded from electrodes (E5 and E6) around the stimulation site. This result could be attributed to enhanced local network formation in the first somatic region by repetitive neural activation through daily optical stimulation^55^. With increasing connectivity between the two somatic regions via the neurite region from DIV 11 to DIV 13 (Supplementary Figs. 22, 23, and 24, and Supplementary Table 4), more vigorous synchronized activities, neuronal firing rates, and increased number of networks occurred in all of the regions at DIV 14 (Fig. 5h–j and Fig. 6g–i). Our 3D high-density MEA also made it possible to measure the extracellular action potentials in the neurite region from DIV 11 to 14, which indicated that the propagation of the action potentials had occurred between the two different neuronal populations^56^ (Supplementary Figs. 21, 23, and 24).

**Fig. 7:**
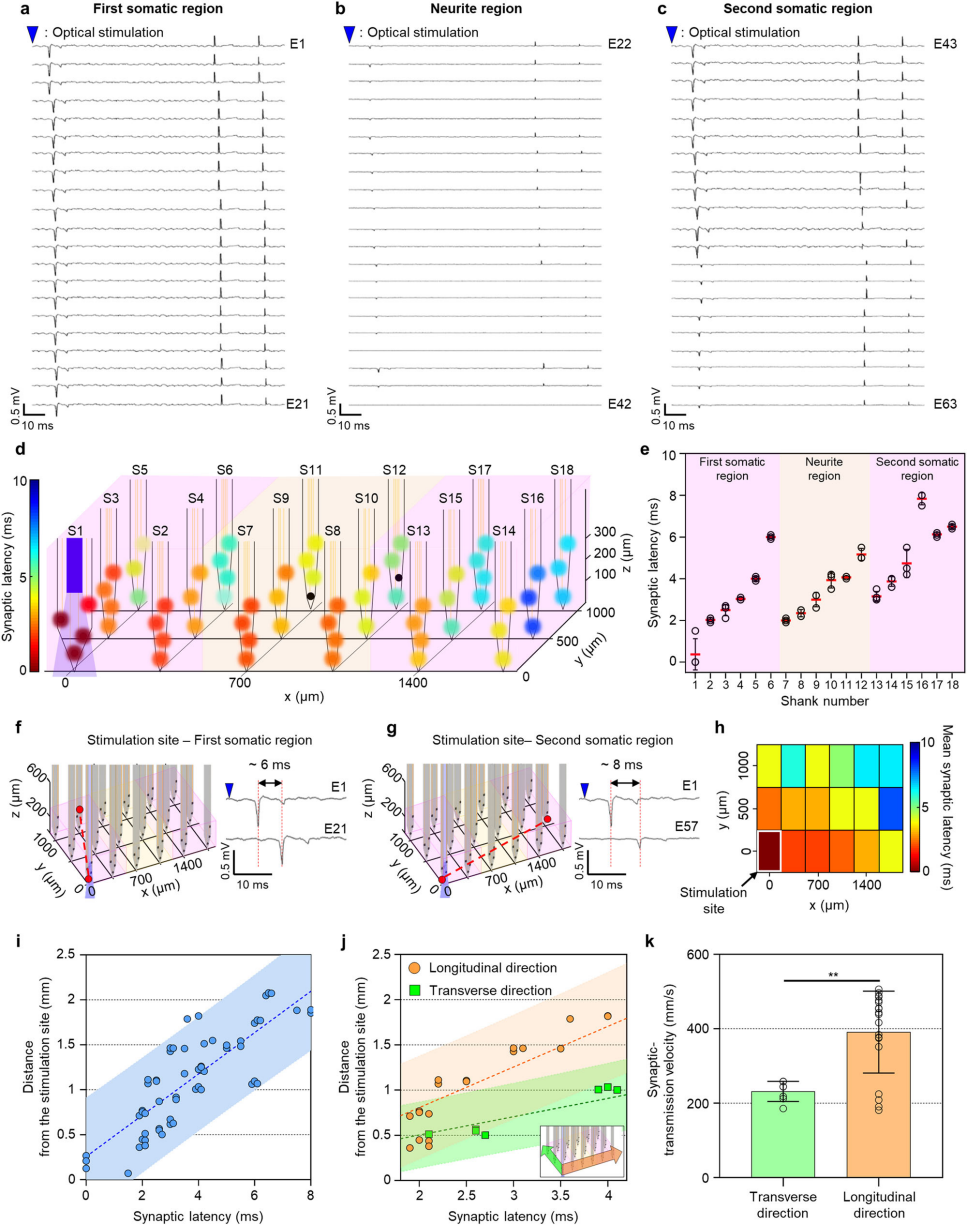
Synaptic latency and transmission velocity between two somatic regions in the compartmentalized two-group 3D neural network model. **a**–**c** Transiently spiking signals of neurons recorded from 63 electrodes of the 3D MEA by optical stimulation in the first somatic (**a**; electrodes 1–21), neurite (**b**; electrodes 22–42), and the second somatic (**c**; electrodes 43–63) regions at DIV 14. The blue triangle indicates the onset time of the optical stimulation. **d** 3D color-mapped synaptic latency at each electrode, relative to the optical stimulation on shank 1. Black-colored circle indicates no signals recorded from the electrodes. The light blue indicates transmitted light from the shank 1. **e** Synaptic latency at each electrode (white circle) in the first somatic (shanks 1-6), neurite (shanks 7–12), and the second somatic (shanks 13–18) regions. The red line indicates mean synaptic latency of the signal recorded from each electrode on each shank. Data are presented as mean values +/− s.d. with individual data points (white circle; *n* = 3 signal recorded electrodes for shank 4, 5, 9, 10, 11, 12, 14, 15, 16, 18 and *n* = 4 signal recorded electrodes for shank 1, 2, 3, 6, 7, 8, 13, 17). **f**–**g** 3D visualization of the shank map from the stimulation site to electrode 21 on shank 6 in the first somatic region (**f**) and electrode 57 on shank 16 in the second somatic region (**g**) (red dotted line in left panel). Transiently spiking signals of neurons recorded from electrodes 1 and 21 (**f**), and from electrode 1 and 57 (**g**) (right panel) to display synaptic latency within the first somatic region from the stimulation site. The blue triangle indicates the onset time of the optical stimulation. The light blue indicates transmitted light from the shank 1. **h** Color-mapped mean synaptic latency at each shank. **i** Scatter plot of distance to all electrodes from the stimulation site depending on synaptic latency. The dotted line and shaded area indicate the best fit of linear regression and the 95% confidence level, respectively. The slope of the linear regression is the synaptic transmission velocity. **j** Scatter plot of distance from the stimulation site to the electrodes along the longitudinal (orange; orange arrow in inset image) and transverse (green; green arrow in inset image) directions depending on synaptic latency. **k** Bar graph showing the synaptic transmission velocity along the transverse and longitudinal directions. Data are presented as mean values +/− s.d. with individual data points (white circle; *n* = 7 signal recorded electrodes for the transverse direction and *n* = 19 signal recorded electrodes for the longitudinal direction).**P = 0.0010. Statistical significance was tested with a two-tailed unpaired *t*-test. Source Data is available as a Source Data file for Fig. 7e, h-k.

Significantly, we were able to analyze the synaptic latency between the two neural groups in a 3D setting at DIV 14 (Fig. 7a–c). The synaptic latency serves as direct evidence of the functional connectivity between the two regions. We estimated the synaptic latency by comparing relative time points of the signals recorded from each electrode with a reference timestamp at the optical stimulation (Fig. 7d). We found that the synaptic latency was shorter than 10 ms, i.e., it ranged from 2 to 8 ms in all three regions with the exception of the stimulation site (Fig. 7d and 7e). However, more delayed synaptic latency was evident in the second somatic region, which indicated that the longer the distance from the stimulation site was, the more delayed the signal became. We confirmed that the most prolonged synaptic latencies in the first and second somatic regions were 6 ms (between electrode 1 on S1 and electrode 21 on S6) and 8 ms (between electrode 1 on S1 and electrode 57 on S16) (Figs. 7f and 7g). Considering the previously reported typical synaptic latency of approximately 1 to 4 ms^57^, we inferred that multiple synapses could connect the neurons near electrodes 21 or 57. Also, signal delays varied along the *z*-axis on some shanks (e.g., shank 15; S15) (Fig. 7d and 7e). This observation suggested that neurons at different positions along the *z*-axis could receive different synaptic inputs. To summarize, the distribution of synaptic latency in each region showed signal delays of approximately 3 to 8 ms between the two neural networks (Fig. 7h).

Interestingly, synaptic transmission along a longitudinal direction (i.e., the xz-plane when y = 0) took longer than that along a transverse direction (i.e., the yz-plane when x = 0) (Fig. 7d and 7h). As an excellent representative, for two locations approximately 1 mm away from the optical stimulation site, the relative mean signal delays were 4 ms on shank 5 (S5) and 2.35 ms on shank 8 (S8) (Fig. 7d). Accordingly, the slope of a linear regression curve in the synaptic latency versus the distance from the optical stimulation can be considered to be the synaptic transmission velocity. We found that the synaptic transmission velocities ranged from 230 ± 42.6 mm⋅s^−1^ irrespective of directions, 449 ± 106 mm⋅s^−1^ along the longitudinal direction, and 202.6 ± 69.8 mm⋅s^−1^ along the transverse direction (Fig. 7i and 7j). The synaptic transmission velocity along the longitudinal direction was about twice as fast as that along the transverse direction (Fig. 7k). These results indicated that the neurons between the two groups were connected in a complex manner via one or multiple synapses.

This difference would result from the neurite region’s presence along the signal propagation path in our 3D culture model. More specifically, the neurite region’s placement, combined with our multifunctional 3D MEA, played a pivotal role in estimating faster transmission velocity along the longitudinal than the transverse direction. The density of synapses in the neurite region was much lower than that in the soma regions. The synaptic latency represents the required time for the signal transmission from the pre-synapse to the post-synapse, which is known to be longer than the signal transmission along neurites (more specifically, axons)^58^. Therefore, we hypothesized that the synaptic transmission velocity would inversely proportional to the synaptic density. In other words, because the number of synapses along the longitudinal path (the neurite region) was much smaller, the longitudinal transmission was significantly less impeded than the transverse transmission.

We compared the synaptic latency along the longitudinal direction between the soma and neurite regions to prove this hypothesis. We confirmed that the longitudinal velocity in the neurite region was faster than that of the somatic regions (Supplementary Fig. 25). Furthermore, we compared the longitudinal and transverse velocities in the first somatic region, where the number of synapses was similar in both directions. We reaffirmed that the synaptic transmission velocity along the two directions was similar (Supplementary Fig. 26).

Overall, our 3D multifunctional MEA enabled the neural network dynamics by the direct confirmation of the functional connectivity between two different populations of neurons cultured in 3D with a high spatiotemporal resolution.

### A further application to human-derived organoids

Organoids are 3D cell aggregates with architecture and functionality similar to those of a living organ through self-renewal and self-organization by stem cells^59^. Therefore, human-derived organoids are in the spotlight as next-generation in vitro model^59,60^. However, functional analysis of human-derived brain or spinal cord organoids has still been mostly performed on a 2D MEA^29,30^, which possesses an inherent limitation for investigating the functional connectivity inside organoids with 3D structures.

To verify our system’s applicability in human-derived organoids, we exploited spinal cord organoids derived from human induced pluripotent stem cells (iPSCs) (Supplementary Fig. 27a–c). Considering the organoids’ size (~700 μm in diameter), which is similar to the size of each somatic or neurite region, we measured neural activities in mature (i.e., 3-month-old) organoid using a needle-type MEA integrated with 16 electrodes and microfluidic channels (Supplementary Fig. 27c). We successfully measured neural activities in the organoid from all the 16 electrodes (Supplementary Fig. 27d–e). Notably, neural signals inside the organoid were synchronized around most electrodes (Supplementary Fig. 27f). This result indicates that neurons in the organoid were inter-connected robustly.

To confirm the functional connectivity and the capability of monitoring temporal evolutions, we injected tetrodotoxin (TTX), which blocks neurons’ sodium channels, through the microfluidic channel of the MEA. Immediately after the TTX injection, the organoid’s neural activities ceased dramatically (Supplementary Fig. 27d and 27g), and accordingly, the robust interconnections between neurons also disappeared (Supplementary Fig. 27h and Supplementary Table 5). This demonstration reveals that our multifunctional MEA system can also be applied readily to 3D organoids to monitor and modulate intra-organoid’s neural activities, which could be further extended to evaluate pharmaceutical candidates’ safety and efficacy with patient-derived organoids.

## Discussion

The accurate modulation capability with a high-density electrode array of the existing neurological tools such as 2D and 3D MEAs has been a significant barrier for analyzing neural circuit dynamics in developing 3D neural tissues in vitro. To overcome this barrier, we developed a miniaturized system with simultaneous capabilities of 3D culture, daily recording, and local stimulations, all integrated within an incubator. Specifically, we presented a 3D high-density multifunctional MEA system with functions that are essential for precise analysis and modulation of neural activities in engineered 3D neural tissues in vitro, including high-density and large-scale electrical recording capability, a 3D volume, local optical stimulation, and drug delivery capabilities. The high-density microelectrodes on the 3D shank array structure enabled the investigation of the dynamics of neural networks formed by neuronal connections in vitro with real-time recording over a two-week culture period. Also, the optical and chemical stimulation capabilities served as an integrated verification tool for functional connectivities between neurons both in a single group and in two compartmentalized neural groups. Notably, we were able to measure the synaptic latency and corresponding synaptic transmission speed within a 3D neural tissue where two different populations of cortical neurons formed functional networks in vitro. Also, our 3D multifunctional MEA inserted 3D neural tissues in vitro would serve as a powerful toolset to study the effects of pharmacological intervention on neural networks, for example, by simultaneously delivering various biochemical factors, such as inter-neuronal receptor agonists and antagonists, through the microfluidic channels and by providing real-time analyses of synaptic activities. These enabling technologies would allow for reconstructing more complex models of neural circuits and enhancing the development of human disease models in vitro.

We have developed the 3D multifunctional MEA primarily for investigating 3D neural circuit dynamics in engineered 3D neural tissues in vitro, which was impossible with our previous 2D multifunctional MEA^26^. The 3D high-density electrode array implemented by applying the stacking method^35^ enabled the precise mapping of functional connectivity between neurons in the entire 3D neural tissue volume. Also, we improved the recording performance, compared with the previous device^26^, by electroplating Pt black on electrodes^36^. Furthermore, we integrated a small LED for optical stimulation, which eliminated external light sources and enabled the system’s use in an incubator. In addition, a two-photon microscopy system is also a useful tool for selectively modulating and measuring neural activities in three dimensions^61^. However, this system has a relatively complex configuration requiring a separate incubation system for applying to 3D in vitro models. In addition, the system not only has a low temporal resolution (30 fps) but also covers a limited field of view (FOV) of 240 × 240 × 300 μm^3^ due to image acquisition through the lens, which is only 3% compared with the measurable volume of our system (1850 × 1000 × 300 μm^3^). Thus, our 3D multifunctional MEA system exhibits more advantageous features for investigating in vitro 3D neural networks’ functionality than previously reported systems.

Also, depending on the 3D in vitro model’s size, at least recording shank array of our 3D MEA can be readily scaled up without further complicating the system. However, the multifunctional shank expansion can serve as a technical hurdle by complicating the overall system. For example, the integration of LED-coupled fiber in each shank requires several optical interfaces. Also, integrating multiple pumps and tubes into probe shanks for independent drug deliveries requires cumbersome fluidic interfaces. These limitations can be overcome by attaching μLED^62,63^ in each shank and applying active valves^64,65^ for on-device fluidic control. In addition, as neurotechnologies continue to advance, our system may still be improved further with a few additional features. For example, a higher number of microelectrodes within the same volume of an engineered neural tissue could enable more sophisticated observations, such as the propagation of axonal action potential^66^. Also, the integration of light source array onto the tip of every shank would allow more complex circuit studies in vitro. Furthermore, the integration of complementary metal-oxide-semiconductor-based (CMOS-based) microelectrode array^67^ or monolithic μLED array^68^ in our 3D MEA configuration would extend useful applicability in 3D brain models in vitro.

Finally, our system can be readily applied to the functional mapping of various neural circuits in vivo, like the previous 2D multifunctional MEA^26^. Applying our 3D MEA to in vivo applications would enable the investigation of complex functional connectivity among three or more brain regions. Furthermore, we can derive extensive neuronal information from the 3D neural models by analyzing the correlation between structural and functional networks using our 3D MEA system and advanced 3D volumetric imaging techniques such as a clearing method^69^. In conclusion, we expect our 3D multifunctional MEA to open up opportunities for studies of neural circuits by investigating functional neuronal networks in 3D neural models, including in vivo.

## Methods

### Fabrication and packaging of the 3D multifunctional MEA

The fabrication of a 3D multifunctional MEA is divided mainly into three steps: (1) the fabrication of three 2D multifunctional MEAs, one of which is to be integrated with the microfluidic channels through the microelectromechanical systems (MEMS) process, (2) the assembly of the 3D multifunctional MEA by stacking the 2D MEAs, and (3) the formation of electrical, fluidic, and optical interfaces.

First, the 2D multifunctional MEAs integrated with the microfluidic channels were fabricated by the previously developed fabrication process^34^. We formed side (25 μm high and 25 μm wide) and center (25 μm high and 10 μm wide) cavities in a four-inch silicon-on-insulator (SOI) wafer with 40 μm-thick top silicon using deep reactive ion etching (DRIE). In a vacuum, the SOI wafer with the cavities was bonded anodically to a 500 μm-thick borosilicate glass wafer (Borofloat^®^33, Schott). The glass wafer was thinned by 100 μm by chemical mechanical polishing (CMP), which was followed by reflowing at 750 °C for 90 min using rapid thermal annealing (RTA; KVR-4000, Korea Vacuum Tech, Ltd.) to fill the cavities partially, which allowed for producing the microfluidic channels underneath the embedded glass layer. After the reflow of the glass, unnecessary glass was removed using the CMP. Next, a 400 nm-thick first passivation layer (SiO_2_) was deposited. Then, a 20 nm-thick titanium (Ti) layer and a 300 nm-thick gold (Au) layer were deposited sequentially, patterned, and etched for the formation of signal lines. Then, the second passivation layer (SiO_2_) was deposited, and microelectrode areas were opened by reactive ion etching (RIE). The microelectrode areas were patterned selectively with Ti and platinum (Pt) by depositing a 20 nm-thick Ti layer and a 150 nm-thick Pt layer, followed afterwards by the lift-off process. After patterning of the top Si layer in the shape of 2D MEA, the structure was released from the backside by DRIE.

Second, the 3D multifunctional MEA was formed by stacking and bonding the 2D MEAs. Each 2D MEA was designed with a different body size to provide sufficient bonding margins; for example, the body of the first layer MEA at the bottom was 3 mm wider than that of the second layer MEA. The second layer MEA was bonded manually on the first layer MEA at the bottom using a fast-curing epoxy under a microscope. Likewise, the third layer was glued to the second layer.

Last, the fabricated 3D multifunctional MEA was packaged to provide fluidic, electrical, and optical interfaces. First, we fabricated a PDMS microfluidic chip as a fluidic interface bridging between the inlet of the MEA and the drug delivery system. A degassed mixture of an elastomer base and a curing agent with a weight ratio of 10:1 was poured into a metal mould with a bottom surface patterned with a fluidic pathway and cured at 80 °C for 1 h. A peeled-off PDMS chip was bonded on the body of the 3D MEA by oxygen plasma using a plasma generator (Covance-MP, Femto Science). Then, the 3D MEA was bonded on a custom PCB using the fast-curing epoxy for electrical and mechanical connections with a microdrive system. The electrical pads on the body of the 3D MEA were wire-bonded to pads on the custom PCB, and two flexible printed circuit (FPC) connectors were soldered for electrical connections between the 3D MEA and the Intan recording system (RHD USB interface board with RHD 64-Channel Recording Headstages, Intan Technologies). We thinned the multimode optical fiber with a 50-μm in diameter core and a cladding layer that was 125 μm in diameter (GIF50, Thorlabs) in 49% [w/w] hydrofluoric acid (HF) solution to a diameter of about 60 μm. The thinned optical fiber was aligned on the shank embedded with the microfluidic channels under a microscope. After aligning the fiber on the shank, the fiber was fixed using an UV-curable epoxy (NOA 148, Norland Products, Inc.). To provide optical stimulations in an incubator, we coupled small blue LED (XQ-E High Intensity LED, Cree, Inc.) at the end of the fiber and filled the gap between the optical fiber and the LED using the UV-curable epoxy as a reflective index matching material. Then, black ink was applied on the UV-curable epoxy to block any light leaking from the LED. Finally, a biocompatible epoxy (EPO-TEK 320, Epoxy Technology, Inc.) was applied at the body of the 3D MEA to protect the bonding wire.

### Characterizations of the 3D multifunctional MEA

To electroplate Pt-black on the Pt microelectrodes to enhance the effective surface areas, we used a mixture of 3% [w/v] hexachloroplatinic acid hydrate (HCPA), 0.025 N HCl, and 0.025% [w/v] lead acetate in deionized (DI) water as an electroplating solution^36^ in which the 3D MEA, a Pt wire, and an Ag/AgCl wire were immersed. With three electrode configurations (i.e., working electrode (WE): 63 Pt microelectrodes; counter electrode (CE): Pt wire; reference electrode (RE): Ag/AgCl wire), Pt-black particles were electroplated selectively on the Pt microelectrodes of the 3D MEA by applying an electrical potential (0.2 V from the CE to WE, 35 s) using a potentiostat (PalmSens3, PalmSens).

After electroplating, we evaluated the functional characterizations of the 3D multifunctional MEA by the measurement setup reported previously^26^. Briefly, to measure impedance on the Pt and Pt-black microelectrodes, electrochemical impedance spectroscopy (EIS) was performed in 1× phosphate-buffered saline (PBS) with a saturated calomel electrode (CHI 151, CH Instruments, Inc). The impedance of the 63 microelectrodes was measured in a frequency sweep mode (10 Hz–10 kHz) using an impedance analyzer (nanoZ, Neuralynx).

We used a pressure-driven drug delivery system for fast response time. To measure flow rates through the embedded microfluidic channels of the 3D MEA, we connected the interfacial PDMS chip on the 3D MEA to mass flow controllers (MFC, National Instruments) through Tygon tubing (ID: 0.5 mm, OD: 1.5 mm; S-54-HL) and a 23-gauge needle. To adjust the input pressure precisely, we connected an electro-pneumatic regulator (ITV0051-2BL, SMC Pneumatics) from a nitrogen tank. By injecting 1× PBS through the microfluidic channels of 3D MEA in three different environments (air, 0.25% [w/v] neuron-seeded collagen, and 0.25% [w/v] cell-free collagen), we measured the distances the liquid moved in the tubing.

To measure the optical power output from the end of the fiber on the 3D MEA, we used a photodetector (918D, Newport, Inc.) coupled with an optical power meter (1936-R, Newport, Inc.). The end of the fiber was placed near the photodetector, and we measured the output power while the LED remained turned on. Fluctuations in measured power were within ±0.002 mW. We used the Monte Carlo simulation to profile the distribution of the light transmitted from the fiber^39,40^. We simulated a collagen scaffold with a domain size of 383 × 250 × 120 (matrix of voxels) and with a voxel size of 0.3 × 0.3 × 0.3 mm^3^. We applied an absorption coefficient of 0.3 (1⋅mm^−1^), a scattering coefficient of 29 (1⋅mm^−1^), the anisotropy of 0.89, and a reflective index of 1.34. A light source was located at voxel (83, 83, 0 matrix of voxels) with a light angle of 21.9°. The light source launched 3.7 × 10^11^ photons⋅ms^−1^, corresponding to 0.15 mW at 473 nm.

### Configuration of the 3D multifunctional MEA system

We devised a miniaturized cubicle for measuring neural activities in the growing 3D neural network model in an incubator. The miniaturized incubating structure consisted of (1) a custom-designed microdrive, (2) a PDMS culture chamber with a well, and 3) an acrylic enclosure. The custom microdrive was fabricated by mechanical machining, and it was composed of moving and supporting parts. The entire structure was made of stainless steel to prevent corrosion of the custom-designed microdrive in a CO_2_ incubator. The custom-designed microdrive had both moving and supporting parts. The moving part had a mover (31 × 5 × 10 mm^3^) with two 1 mm holes to fix the 3D MEA, a 20 × 1.5 mm screw in the center for controlling the height of the mover, and two supporting cylinders on both sides. The maximum working distance of the mover was 20 mm, and one revolution of the screw allowed a 0.3 mm vertical movement. The supporting part was used to tightly fix the moving part and integrate the culture chamber with the 3D MEA. The culture chamber was made of PDMS, the volume of the cell culture was 2.5 × 1.5 × 0.5 mm^3^, and the overall size was 20 × 20 × 10 mm^3^. The culture chamber was positioned below the 3D MEA during horizontal alignment on a microscope, followed by gluing with uncured PDMS. This step was essential to locate the 3D MEA in the compartmentalized culture area. In particular, we placed six shanks per each area (i.e., two somatic regions and a neurite region) separated each by a PET film. And then, we precisely controlled the position of the PDMS chamber with a resolution of better than 50 μm by pushing the chamber using a linear actuator (M-561D, Newport) with a custom structure along *x*-axis and *y*-axis on the microscope. Also, to precisely control the height of 3D MEA in the 3D neural network model, we lowered the MEA using the custom microdrive under the microscope until it touched the bottom surface of the culture chamber and bent slightly. And then, we slowly raised the MEA until it was fully straightened. As a result, the tip of the 3D MEA was in contact with the bottom surface of the culture chamber. The acrylic enclosure (10 × 8 × 8 cm^3^) prevented undesirable evaporation of the culture medium and contamination, and it was large enough to accommodate the custom microdrive with the culture chamber and the 3D MEA. Top and bottom holes were used to insert the FPCB cable and to fix the microdrive, respectively. Two holes (1 mm in diameter) were drilled through each side of the enclosure for supplying O_2_ and CO_2_.

### Staining of collagen microfibrils

To observe distributions of collagen microfibrils when the 3D MEA was inserted before or after the collagen loading, we stained the collagen microfibrils with 5-(and-6)-carboxytetramethylrhodamine succinimidyl ester (TAMRA; Invitrogen) as reported previously^13^. Briefly, 5 μM TAMRA was mixed in 1× PBS and was applied to the culture chamber. After incubating at room temperature for 1 h, the culture chamber was rinsed three times with 1× PBS. On a confocal laser scanning microscope (LSM 800, Carl Zeiss), we observed stained collagen microfibrils near a shank of 3D MEA and *z*-stacked images (stack size: 25 μm; step size: 1 μm).

### Preparation of two types of models of 3D neural networks

Pregnant Sprague Dawley (SD) rats (embryo 18; E18) were purchased (DBL Co., Ltd.) and sacrificed for 3D neural cultures. We followed a previously reported protocol^13^ for harvesting the rats’ primary cortical neurons. Briefly, embryos from the pregnant SD rats were decapitated, and the cerebral cortex was dissected and removed. The cerebral tissue that was extracted was treated with papain solution to dissociate the cells. Then, the dissociated cells were counted in order to calculate the volume of the cell suspension that would be required for the desired seeding density in collagen. Then, 0.5 mL of collagen solution (2.5 mg⋅mL^−1^) was prepared in an ice bucket. Specifically, 133 μL of a collagen stock (354249, Collagen type I rat tail high concentration, 9.40 mg⋅mL^−1^, CORNING) were transferred to a 1.5 mL microtube, and 50 μL of 10× Dulbecco Modified Eagle Medium (DMEM; Sigma-Aldrich) and 207 μL of 1× DMEM were added sequentially, and then the compounds were mixed thoroughly. For the cell-free collagen, 307 μL of 1× DMEM were added. Then, 10 μL of 0.5 N NaOH were added to neutralize the solution, i.e., make the pH ~7. 100 μL of the cell suspension were added to reach the seeding density of 4 × 10^7^ cells⋅mL^−1^ for similar cell density to in vivo^41^, and this was followed by gentle mixing. In the case of the model of the single-group 3D neural network, neuron-seeded collagen was loaded in the cell culture well and then gelated completely in the CO_2_ incubator at 37 °C for 30 min. For the two-group 3D neural network model, two 125 μm-thick polyester (PET) films were inserted into grooves in the cell culture region to create three temporary compartments. The cell-free collagen was loaded in the central compartment, and it was gelated partially in the CO_2_ incubator at 37 °C for 20 min. Then, the neuron-seeded collagen was loaded in the side compartments and was gelated in the CO_2_ incubator at 37 °C for 20 min. Finally, the PET films were removed carefully, and 1.5 mL of the culture medium, consisting of neurobasal Plus medium supplemented with 2% [v/v] B27 Plus supplement (Invitrogen), 2 mM Glutamax-I (GIBCO) and 1% [v/v] penicillin-streptomycin (P/S; GIBCO), were applied in the cell culture chamber. Half of the medium was replaced with a fresh medium after two days for uninfected cell culture (Figs. 2–3 and Supplementary Figs. 9–15). Subsequently, the medium was replaced entirely with a fresh medium daily to provide sufficient nutrients for the cells. For ChR2-infected culture (Figs. 4–7 and Supplementary Figs. 16–26), the culture medium was fully replaced after one day with a fresh medium that contained 5 μL of AAV-EF1α-hChR2(H134R)-eGFP virus (1.25 × 10^12^ GC⋅mL^−1^, KIST Virus Facility). After two more days, half of the medium was replaced with a fresh medium. Subsequently, the medium was replaced entirely with a fresh medium daily to provide sufficient nutrients for the cells. We observed ChR2-infected neurons on a confocal laser scanning microscope (LSM 800, Carl Zeiss) at DIV 6 and DIV 14, emitting green fluorescence, and *z*-stacked images were acquired (stack size: 25 μm; step size: 1 μm).

In this work, we cultured for up to 14 days to observe the changes in connectivity among neurons according to maturation because we started observing cell death after 14 days due to the lack of nutrient supply. Because of both the higher cell seeding density than other typical 3D in vitro neural models^13,15,22,23^ and continuous proliferation of small portions of non-neuronal cells (e.g., glia), included during the isolation of primary neurons from embryonic brains, we needed to change the culture medium more frequently for sufficient supply of nutrients after 14 days. We note that the 3D culture for up to 14 days was sufficient for synapse formation. However, the culture period could be more extended by lowering the cell seeding density in collagen for 28–60 days, as previously reported^13^.

### Generation of human spinal cord organoids

The generation of human spinal cord organoids (hSCOs) was performed according to the previously described protocol^70^. Briefly, hiPSC colonies were treated with SB431542 (10 μM, TOCRIS, 1614) and CHIR99021 (3 μM, SIGMA, SML1046) for 3 days to induce caudal neural stem cells^71^. On day 3 of chemical treatments, the colonies were gently detached from dish and allowed to form neural spheroids in the medium supplemented with basic fibroblast growth factor (bFGF) for 4 days. Subsequently, neural spheroids were cultured for additional 8 days with medium containing retinoic acid (RA) without bFGF. After then, neural spheroids were progressively matured into spinal cord-like organoids in the maturation medium (1:1 mixture of DMEM/F-12 and neurobasal medium (Life Technologies, 21103-049); the media contained 0.5% N2, 2% B27, 0.5% NEAA, 1% P/S, 0.1% β-mercaptoethanol, 1% GlutaMAX (Life Technologies, 35050-061), and 0.1 μM RA).

### Live/dead cell viability assay

Cell viability was assessed from samples of the single-group 3D neural model at DIV 14. The samples were submerged in 1× PBS containing 0.5 μg⋅mL^−1^ calcein-acetoxymethyl (calcein-AM; Sigma-Aldrich) and 2 μg⋅mL^−1^ propidium iodide (PI; Sigma-Aldrich) in the CO_2_ incubator at 37 °C for 30 min. After washing with 1× PBS three times for 30 min each time, we acquired *z*-stacked images (stack size: 50 μm; step size: 1 μm) that had live (green-fluorescent) and dead (red-fluorescent) cells on the confocal laser scanning microscope (LSM 800, Carl Zeiss).

### Immunofluorescence staining and imaging

To visualize the structural connectivity of the single-group and two-group 3D neural network models at DIV 3, 6, and 14, we stained neurites with neuron-specific class III beta-tubulin (mouse anti-Tuj-1, 1:200, T8678, Sigma-Aldrich) antibody and astrocyte with glial fibrillary acidic protein (chicken anti-GFAP, 1:200, AB5541, Sigma-Aldrich) antibody. First, the samples were fixed in 4% [w/v] paraformaldehyde (PFA) in 1× PBS for 4 h at room temperature on a shaker. After washing with 1× PBS five times, each for 30 min, the samples were blocked in a blocking solution that contained 0.1% [v/v] Triton X-100 and 3% [w/v] bovine serum albumin (BSA) in 1× PBS for 24 h at 4 °C on the shaker. After washing with 1× PBS three times, each for 30 min, the samples were incubated with the primary antibodies (Tuj-1 and GFAP) in the blocking solution for 48 h at 4 °C on the shaker. After washing with 1× PBS five times, each for 30 min, the samples were incubated with secondary antibodies (goat anti-mouse conjugated Alexa Fluor 488, 1:200, A-11001, Invitrogen; goat anti-chicken conjugated Alexa Fluor 647, 1:200, ab150171, Abcam) in the blocking solution for 24 h at 4 °C on the shaker. After washing with 1× PBS five times, each for 30 min, the samples also were incubated with 4’,6-diamidino-2-phenylindole (DAPI; 1:1000, D1306, Invitrogen) in the blocking solution for 6 h at room temperature on the shaker. Finally, after washing with 1× PBS five times, each for 30 min, the samples were mounted on glass slides. Fluorescence images were acquired through a ×20 objective lens on the confocal laser scanning microscope (LSM 800, Carl Zeiss). 3D images of the two-group 3D neural network model were acquired by rendering *z*-stacked (stack size: 75 μm; step size: 1 μm) and tile-scanned images.

To visualize neuronal and non-neuronal population in human-derived spinal cord organoid, we fixed the organoids in 4% [w/v] PFA in 1× PBS at 4 °C overnight on a shaker. After washing with 1× PBS five times, each for 10 min, the fixed organoids were then incubated in 30% [w/v] sucrose in 1× PBS at 4 °C on the shaker. After freezing on dry ice, each organoid was sectioned to 40-μm thickness. The sliced organoids were incubated in a blocking solution that contained 0.2% [v/v] Triton X-100 and 3% [w/v] BSA in 1× PBS at room temperature for 1 h on the shaker. Then, the sliced organoids were incubated with the following primary antibodies in the blocking solution at 4 °C overnight on the shaker.: NeuN (rabbit anti-NeuN, 1:1000, ABN78, Millipore), microtubule-associated protein 2 (MAP2; chicken anti-MAP2, 1:5000, AB5543, Millipore), neurofilament-m (NF-M; mouse anti-NF-M, 1:250, 2H3, DSHB), and astrocyte with glial fibrillary acidic protein (GFAP; rat anti-GFAP, 1:500, 13-0300, Invitrogen). After washing with 1× PBS five times, each for 10 min, the primary antibody-conjugated organoids were incubated with the following secondary antibodies in the blocking solution for 30 min at room temperature on the shaker: donkey anti-rabbit conjugated Cy3, 1:500, 711-165-152, Jackson; donkey anti-chicken conjugated Alexa Fluor 488, 1:500, 703-545-155, Jackson; donkey anti-mouse conjugated Alexa Fluor 488, 1:500, A21202, Invitrogen; donkey anti-rat conjugated Cy3, 1:500, 712-166-150, Jackson. After washing with 1× PBS five times, each for 10 min, the secondary antibody-conjugated organoids were also incubated with Hoechest33342 (1:1000) in the blocking solution at room temperature for 30 min on the shaker. Finally, after washing with 1× PBS five times, each for 10 min, the immunostained organoids were mounted on glass slides. Fluorescence images were acquired through a ×25 objective lens on a confocal laser scanning microscope (TCS SP8, Leica).

### Electrophysiology

We measured the neural activity daily from the single-group and two-group 3D neural network models shortly before replacing the culture medium with fresh medium because neurons often stop firing for several hours after the medium are exchanged^72^. We recorded the neural activity every day for 10 min to record spontaneous activity (the first 5 min) and activated signals by local optical stimulation (the next 5 min). All electrophysiological recordings were performed in the CO_2_ incubator at 37 °C through an RHD USB interface board with the RHD 64-channel recording headstage. The recorded signals were filtered and digitized through the Intan USB interface board software (20 kS⋅s^−1^ per channel, 300 Hz high pass filter, 6 kHz low pass filter).

For optical stimulation to the 3D neural model, we used a custom-designed LED drive that adjected the stimulation cycle and pulse width through slide switches. The custom LED drive was connected to the stimulation pin of the 3D MEA with thin electrical wire for light delivery through LED-coupled fiber and to the digital input port of the RHD USB interface board for reading the stimulation time. The applied stimulation frequency was 0.2 Hz, and the pulse width was 2.5 s with a duty cycle of 50%. For the chemical stimulation to the 3D neural model, we used a pressure-driven drug delivery system, an USB-powered small power supply (Analog Discovery 2, Digilent, Inc.), and an 11.1 V Li–Po battery. The small power supply and the battery were connected to the electro-pneumatic regulator of the pressure-driven drug delivery system for pressure control and power supply. Also, the small power supply was connected to the digital input port of the RHD USB interface board for reading the injection time. Using the settings above, the drugs were injected into the 3D neural culture through the microfluidic channels of the 3D MEA. In Fig. 4, after observing the response of neurons by optical stimulation, we injected 1 μL of culture medium mixed with 20 μM CNQX (0190, Tocris Bioscience) and 50 μM AP5 (0106, Tocris Bioscience) at a flow rate of 0.25 μL min^−1^ for 4 min. After 30 min for additionally sufficient diffusion of CNQX/AP5 in an incubator, we resumed recording neural signals with optical stimulation to observe neural activities’ changes. And then, to remove the remaining CNQX/AP5 in the culture chamber, the culture medium was entirely replaced with fresh medium three times in a fume hood. Furthermore, we waited for 1 h to stabilize the neurons. After removing CNQX/AP5, we repeated recording neural signals with optical stimulation to observe the recovery of neural activities.

To monitor neural activities in human-derived spinal cord organoid, we fixed a customized PDMS chamber (diameter of 30 mm and height of 10 mm) with a groove (diameter of 1 mm and height of 0.3 mm) on the bottom plate of the microdrive. After placing the mature (3 month-old) organoid in the groove under a microscope, we slowly inserted the MEA into the organoid using the microdrive. Once inserted correctly, we fixed the microdrive on the acrylic enclosure and measured neural activities in the organoid inside an incubator. After measuring spontaneous activities for 3 min, we injected 6 μM TTX through the microfluidic channels at a flow rate of 0.25 μL min^−1^ for 3 min to suppress the neural activities. After the TTX injection, we continued measuring the neural activities for additional 3 min to observe temporal evolutions.

### Signal analysis

Previous reported MATLAB spike-sorting algorithm^26^ was used to detect neural spikes. We calculated the signal-to-noise ratio (SNR) by dividing the mean of the peak amplitude by the standard deviation of the background noise. Then, we set an amplitude threshold that exceeded three times the level of the noise (approximately 50 μV) and extracted neural signal data. Each signal was displayed on a raster plot, a color-mapped raster plot, and a 2D/3D electrode map. Also, each signal was counted and displayed on a bar plot. Burst activity was analyzed using the ISI_N_-threshold method^73^ with an ISI threshold of 0.1 s and a minimum number of spikes per burst of 3. All statistical analyses were evaluated by the Student’s *t*-test using GraphPad Prism.

We analyzed the synchronized scores between the electrodes and networks based on a previously reported method^32^. Synchrony between electrodes was analyzed using Pyspike. Briefly, the closer the spikes from the two electrodes matched, the closer the synchronized score was to 1, which represents a high degree of synchrony. Conversely, the greater the mismatch was between the spikes from the two electrodes, the closer the synchronized score was to 0, which represents a high degree of asynchrony. For visualization of the community among neurons, we represented the network among the electrodes by the Louvain algorithm using a custom code. We set the electrode as a node (e.g., circles in the network map), and we set the degree of synchronization between the electrodes as an edge (e.g., lines in the network map). We matched the position of the node with the position of the actual electrode. Also, the links with synchronized scores less than 0.5 were filtered out. Nodes with the same color (i.e., electrodes) represented the same community network. The color-mapped synchronization score ranged from 0 (blue) to 1 (red). In addition, the larger the number of connected electrodes becomes, the larger the size of the node becomes.

### Ethical statements

All procedures except human-derived pluripotent stem cell (PSC) related experiments were conducted according to the animal welfare guidelines approved by the Institutional Animal Care and Use Committee of the Korea Institute of Science and Technology. The human PSC related experiment was approved by the Korea University Institutional Review Board.

### Statistical analysis

All statistical analyses were performed in MATLAB (Math Works), Python (Python Software Foundation), or GraphPad Prism (GraphPad Software), using the Student’s *t*-test.

### Reporting summary

Further information on research design is available in the Nature Research Reporting Summary linked to this article.

## Supplementary information

Supplementary Information

Supplementary Movie 1

Supplementary Movie 2

Supplementary Movie 3

Supplementary Movie 4

Supplementary Movie 5

Reporting Summary

## Data Availability

The authors declare that all data supporting the findings of this study are available within the article and its supplementary information files or from the corresponding author upon reasonable request. Source data are provided with this paper.

## Source data

Source data

## Code availability

The data analysis for this study mainly performed using Python 3.7 and the open source packages Py spike and python-louvain, which are available at https://github.com/mariomulansky/PySpike/ and https://github.com/taynaud/python-louvain/. Custom code used for visualization of 3D network maps is freely available at https://github.com/Hyogeun-Shin/Visualization-of-3D-network-map/tree/v1.0.0 (10.5281/zenodo.4306072).
