# Supplementary material for: 3D high-density microelectrode array with optical stimulation and drug delivery for investigating neural circuit dynamics
Source: Nat Commun. 2021 Jan 21;12:492. doi: 10.1038/s41467-020-20763-3 (PMC7820464; doi:10.1038/s41467-020-20763-3)
Supplement: Supplementary file 1 — Supplementary Information [file 41467_2020_20763_MOESM1_ESM.pdf]

Supplementary Information:

3D high-density microelectrode array with optical stimulation and drug delivery for investigating neural circuit dynamics

Shin et al.

DIV	6	7	8	9	10	11	12	13	14
Total number of connections	0	0	10	20	158	301	356	512	1,282

Supplementary Table 1: Total number of connections within 3D network maps based on spontaneous activities in the single-group 3D neural network model (Related figures: Fig. 3a-e and Supplementary Fig. 13).

DIV	6	14
Total number of connections	1	860

Supplementary Table 2: Total number of connections within 3D network maps based on neural activities by optical stimulation in the single-group 3D neural network model (Related figure: Fig. 4e).

	Before injection	After injection	After wash-out
Total number of connections	529	65	400

Supplementary Table 3: Total number of connections within 3D network maps based on neural activities by optical stimulation before and after CNQX/AP5 injection, and wash-out CNQX/AP5 in the single-group 3D neural network model (Related figure: Fig. 4l-n).

DIV	6	7	8	9	10	11	12	13	14
Total number of connections	1	1	2	55	144	524	909	1,076	1,301

Supplementary Table 4: Total number of connections within 3D network maps based on neural activities by optical stimulation in the compartmentalized two-group 3D neural network model (Related figures: Fig. 6d-i and Supplementary Fig. 19).

	Before injection	After injection
Total number of connections	120	0

Supplementary Table 5: Total number of connections within 2D network maps based on spontaneous activities before and after CNQX/AP5 injection in the human-derived spinal cord organoid (Related figure: Supplementary Fig. 27).

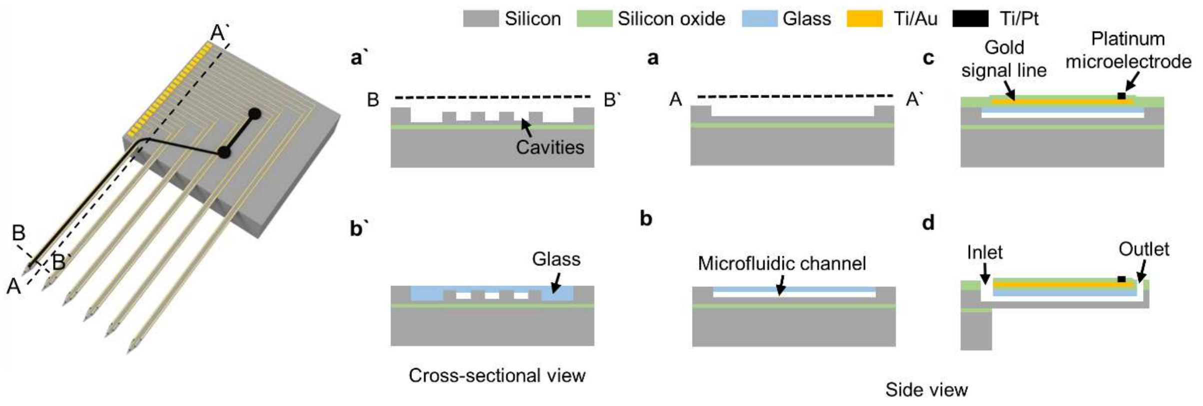

Supplementary Figure 1: Fabrication process of the 2D multifunctional MEA: a Formation of cavities in a silicon-on-insulator (SOI) wafer by deep reactive ion etching (DRIE) process; **b** Formation of microfluidic channels by successive anodic bonding, glass thermal reflow, and chemical mechanical polishing (CMP) process; **c** Formation of signal lines and Pt microelectrodes by successive deposition and etching process; **d** Formation to shank array structure integrated with the microfluidic channels by successive DRIE process.

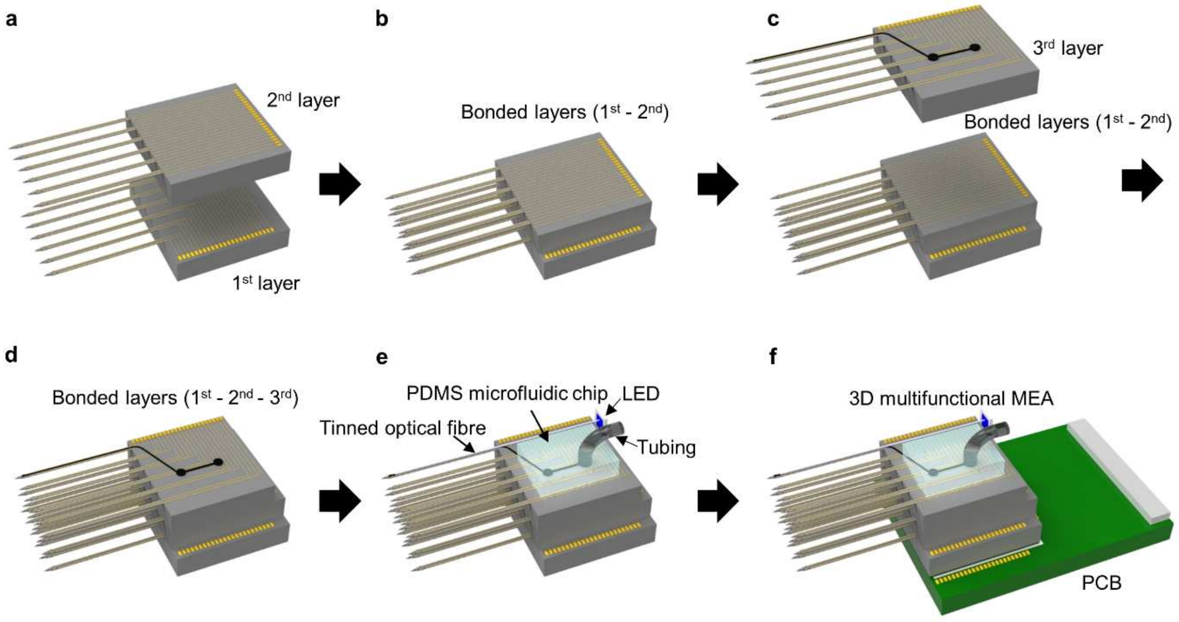

Supplementary Figure 2: Packaging process of the 3D multifunctional MEA: a-d Successive stacking and bonding between layers using fast-curing epoxy under a microscope for formation of 3D multifunctional MEA; **e** Packaging of other components (e.g., tinned optical fibre, PDMS microfluidic chip, LED) for providing fluidic and optical interfaces; **f** Wire-bonding to pads on the custom PCB and soldering of two FPC connectors for electrical connections between 3D MEA and recording system.

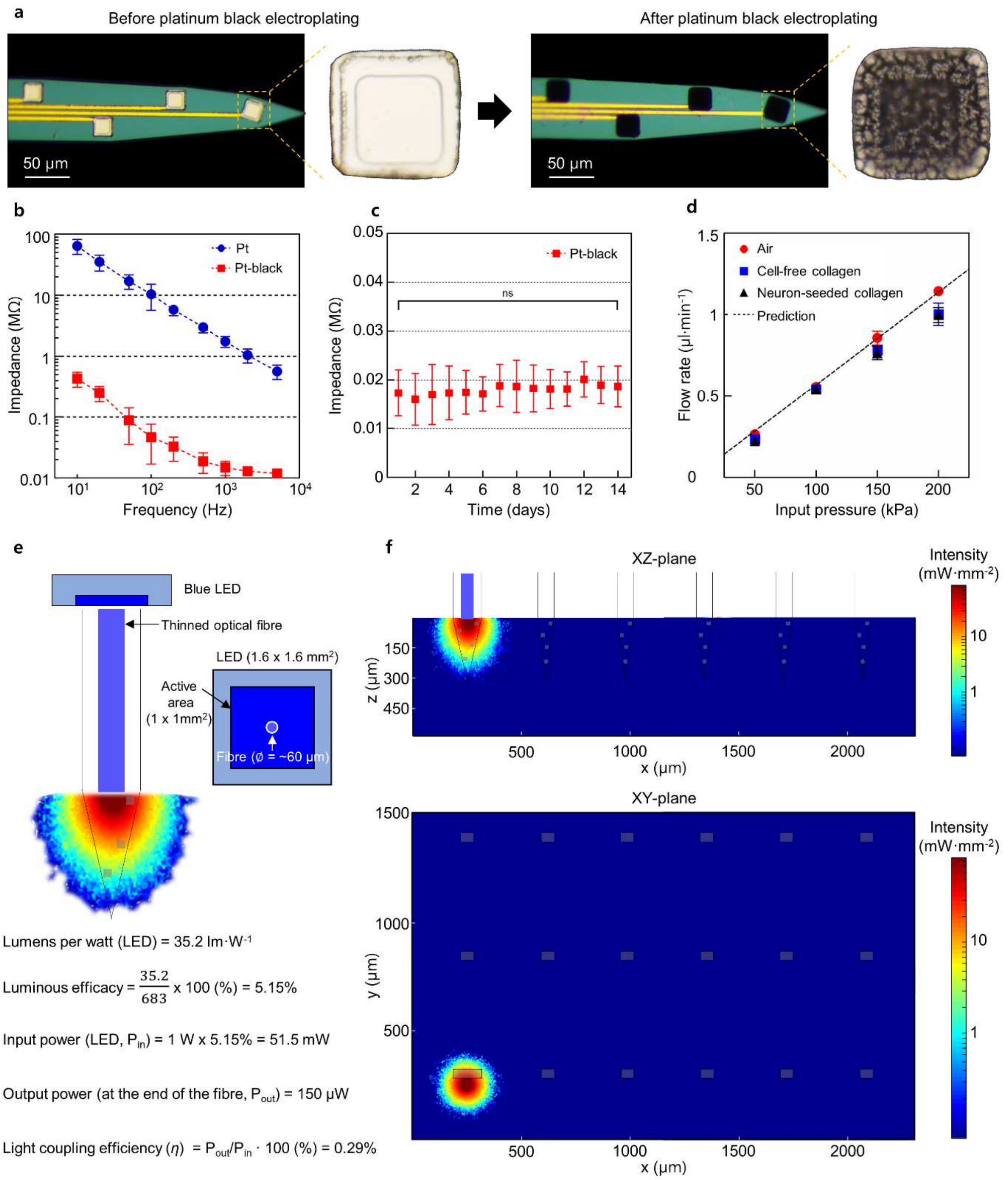

Supplementary Figure 3: Characterizations of the 3D multifunctional MEA: a Representative optical image of a recording shank with Pt and Pt-black electrodes before and after electrodeposition. Pt black electrodeposition were independently repeated at least ten times with similar results to ensure reproducibility. **b** Electrical impedance of all the 63 microelectrodes before (blue circle) and after electrodeposition of Pt-black (red square). Data are presented as mean values +/- s.d., n = 63 electrodes. **c** Electrical impedance of the 63 Pt-black microelectrodes at 1 kHz for 14 days. Data are

presented as mean values +/- s.d., ns P = 0.1012 (day 1 – 14), n = 63 electrodes. Statistical significance was tested with a two-tailed unpaired t-test. **d** Flow rates through microfluidic channels in the air (red circle), cell-free collagen (blue square), and neuron-seeded collagen (purple triangle) from 3 devices. Data are presented as mean values +/- s.d. **e** Schematic diagram showing light transmission from the LED to the thinned optical fibre, and the calculation on light coupling efficiency from the LED to the thinned optical fibre. **f** Simulated distribution of the transmitted light in collagen using the Monte-Carlo simulation.

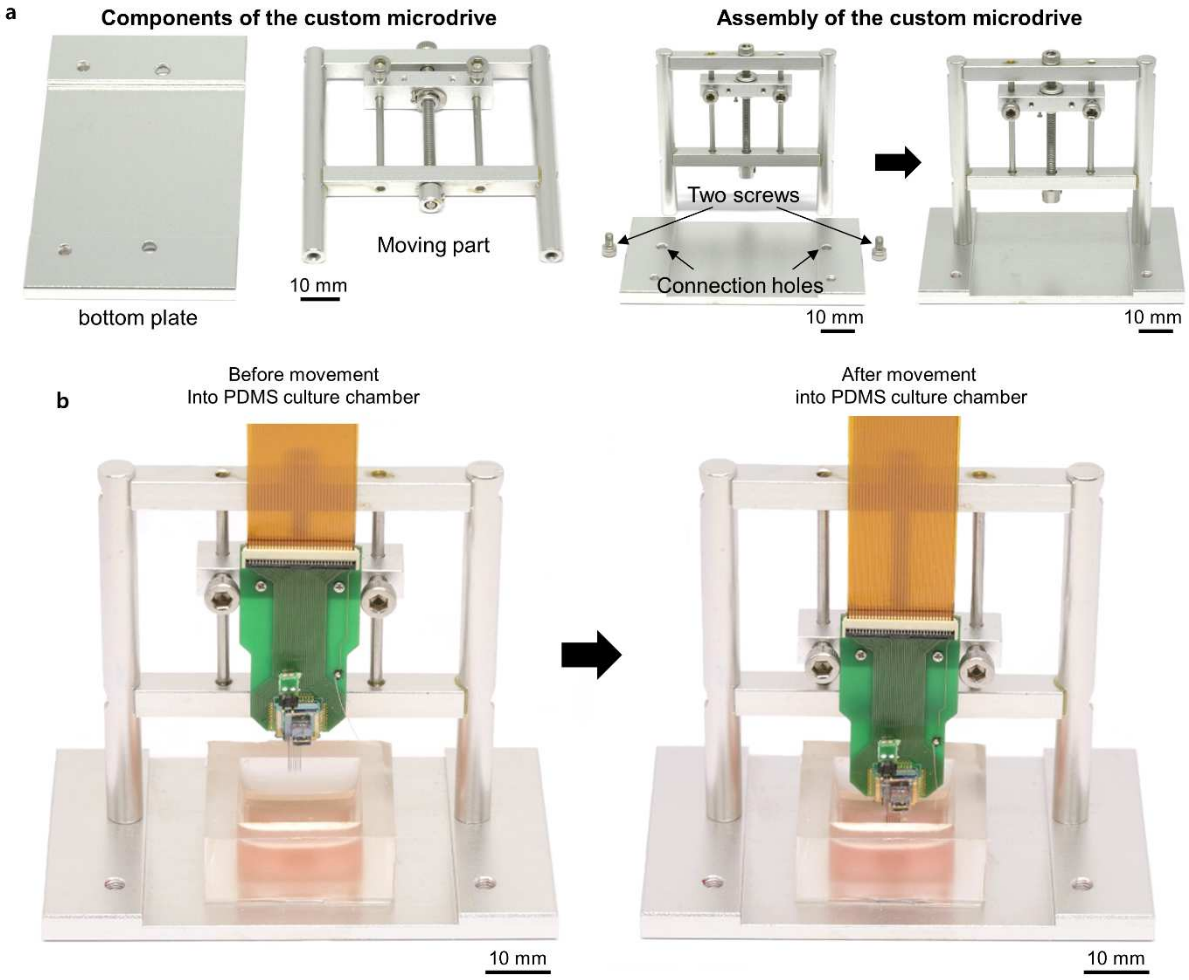

Supplementary Figure 4: Photographs of the custom microdrive: a Photograph of the components of the custom microdrive and the successive photographs showing the assembly of the custom microdive. **b** Successive photographs before and after movement into PDMS culture chamber using custom-designed microdrive.

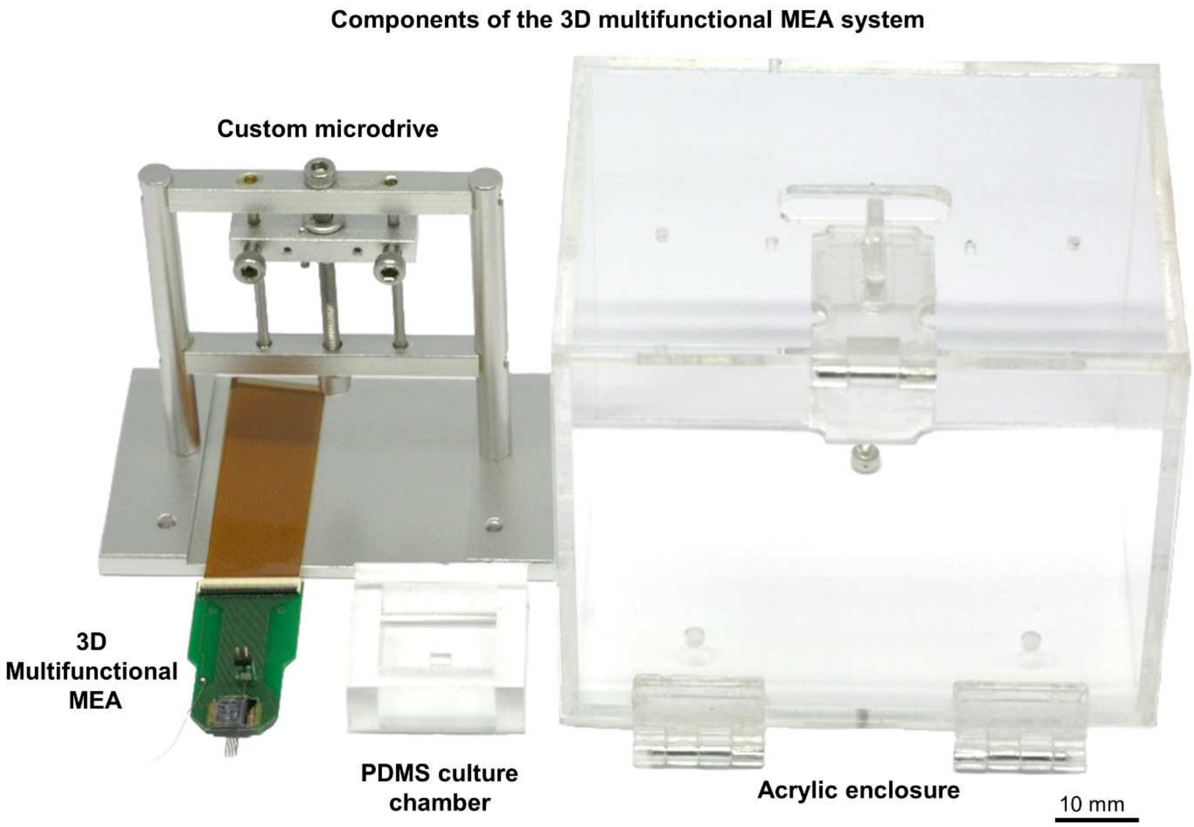

Supplementary Figure 5: Components of the overall 3D multifunctional MEA system: 3D multifunctional MEA, custom microdrive, PDMS culture chamber, and acrylic enclosure.

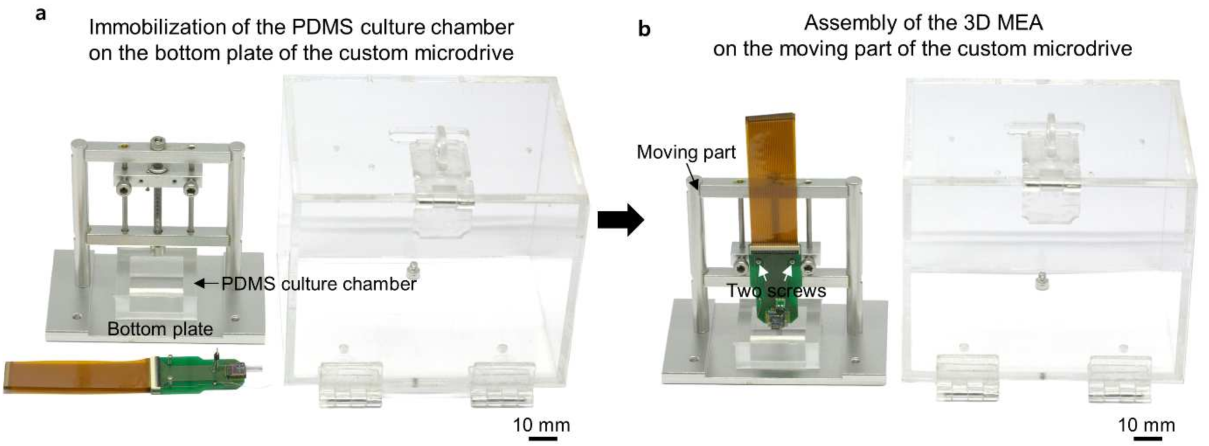

Supplementary Figure 6: Successive photographs showing assembly of the 3D multifunctional MEA system: a Attached PDMS culture chamber on the bottom plate of the custom microdrive using liquid PDMS; **b** Attached 3D multifunctional MEA on the moving part of the custom microdrive by tightening two screws.

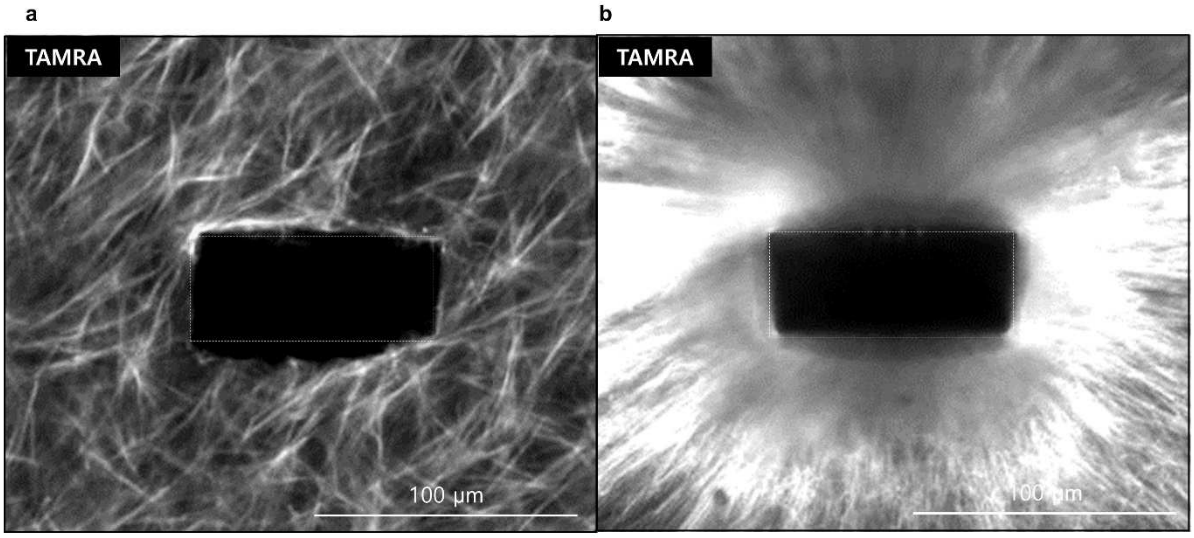

Supplementary Figure 7: Morphology of TAMRA-labeled collagen microfibrils near a shank. a Representative morphology of collagen microfibrils when the 3D MEA was inserted before the collagen loading; **b** Representative morphology of collagen microfibrils when the 3D MEA was inserted after the collagen loading. This experiment was independently repeated at least three times with similar results to ensure reproducibility.

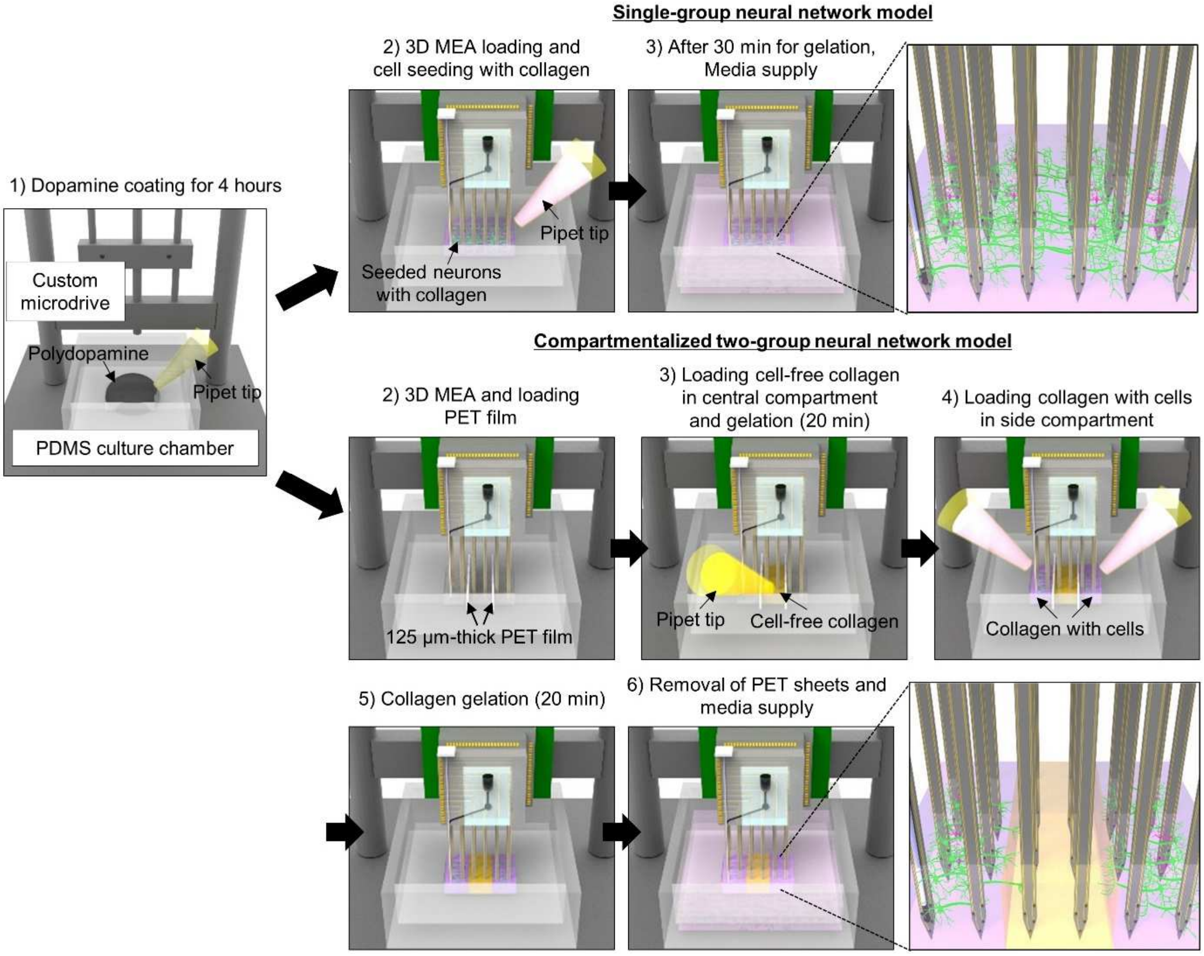

Supplementary Figure 8: Cell culture process for developing two types of 3D neural network models: single-group neural network and compartmentalized two-group neural network.

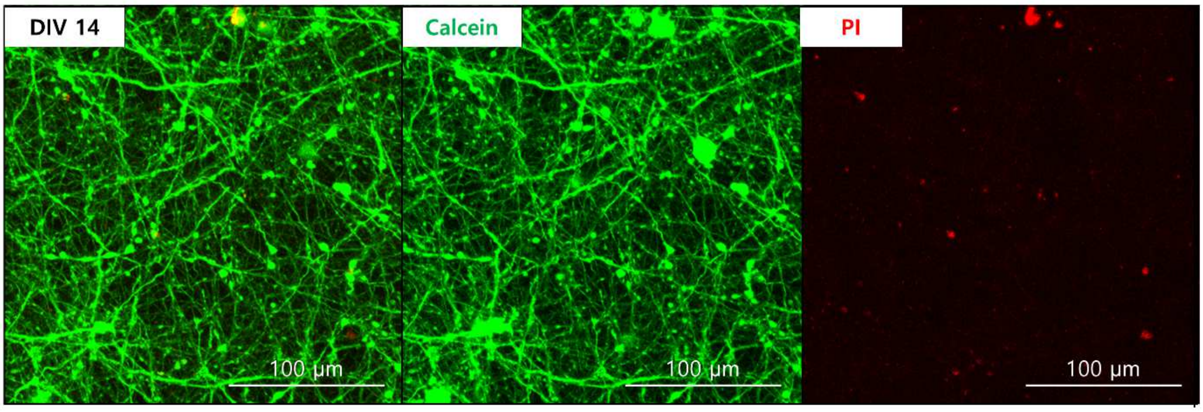

Supplementary Figure 9: Representative confocal fluorescence images of live (green-fluorescent) and dead (red-fluorescent) cells stained with calcein-AM and PI in the single-group 3D neural network model at DIV 14 for observing cell viability. Cell viability assay was independently repeated at least three times with similar results to ensure reproducibility.

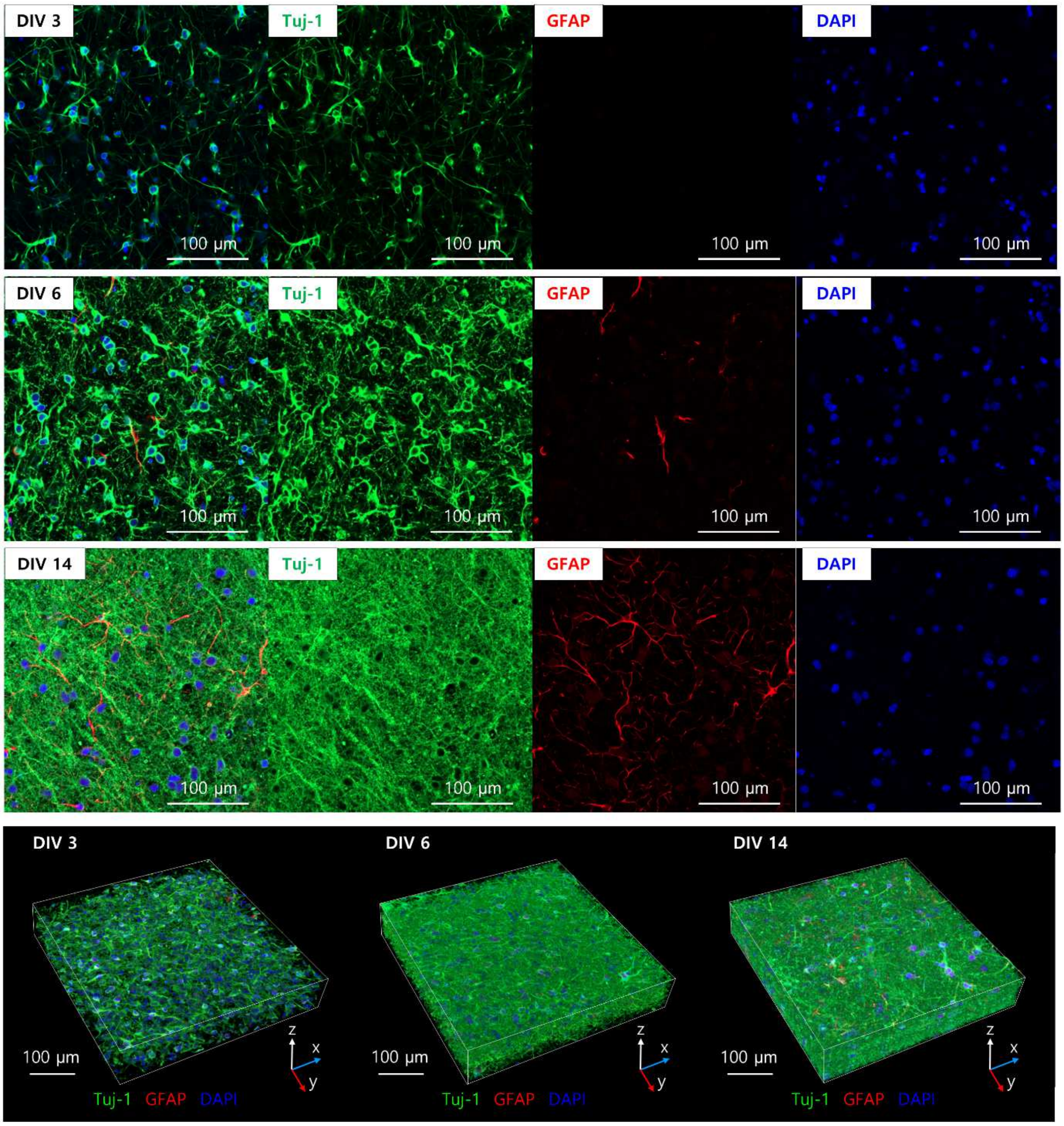

Supplementary Figure 10: Representative 2D and 3D rendered confocal fluorescence images of immunostained neurites (Tuj-1, green-fluorescent), astrocyte (GFAP, red-fluorescent), and cell nucleus (DAPI, blue-fluorescent) in the single-group 3D neural network model at DIV 3, DIV 6 and DIV 14. Immunostaining and imaging were independently repeated at least three times with similar results to ensure reproducibility.

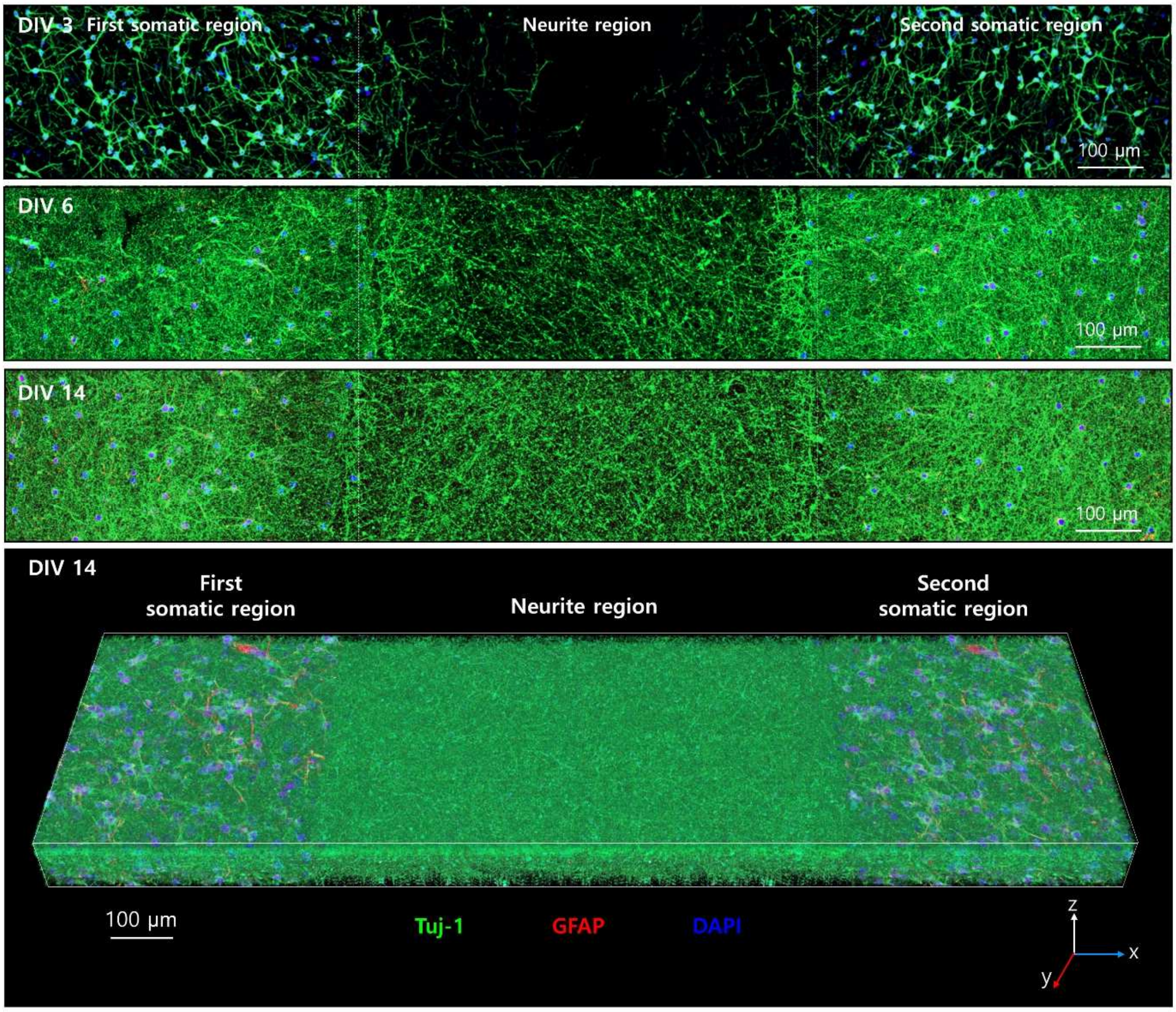

Supplementary Figure 11: Representative confocal fluorescence images showing the structural connection between two somatic regions by the axonal growth in the compartmentalized two-group 3D neural network model: 2D confocal fluorescence images of immunostained neurites (Tuj-1, green-fluorescent), astrocyte (GFAP, red-fluorescent), and cell nucleus (DAPI, blue-fluorescent) at DIV3, DIV 6 and DIV 14, and 3D rendered confocal fluorescence image of the two-group 3D neural network model at DIV 14. Immunostaining and imaging were independently repeated at least three times with similar results to ensure reproducibility.

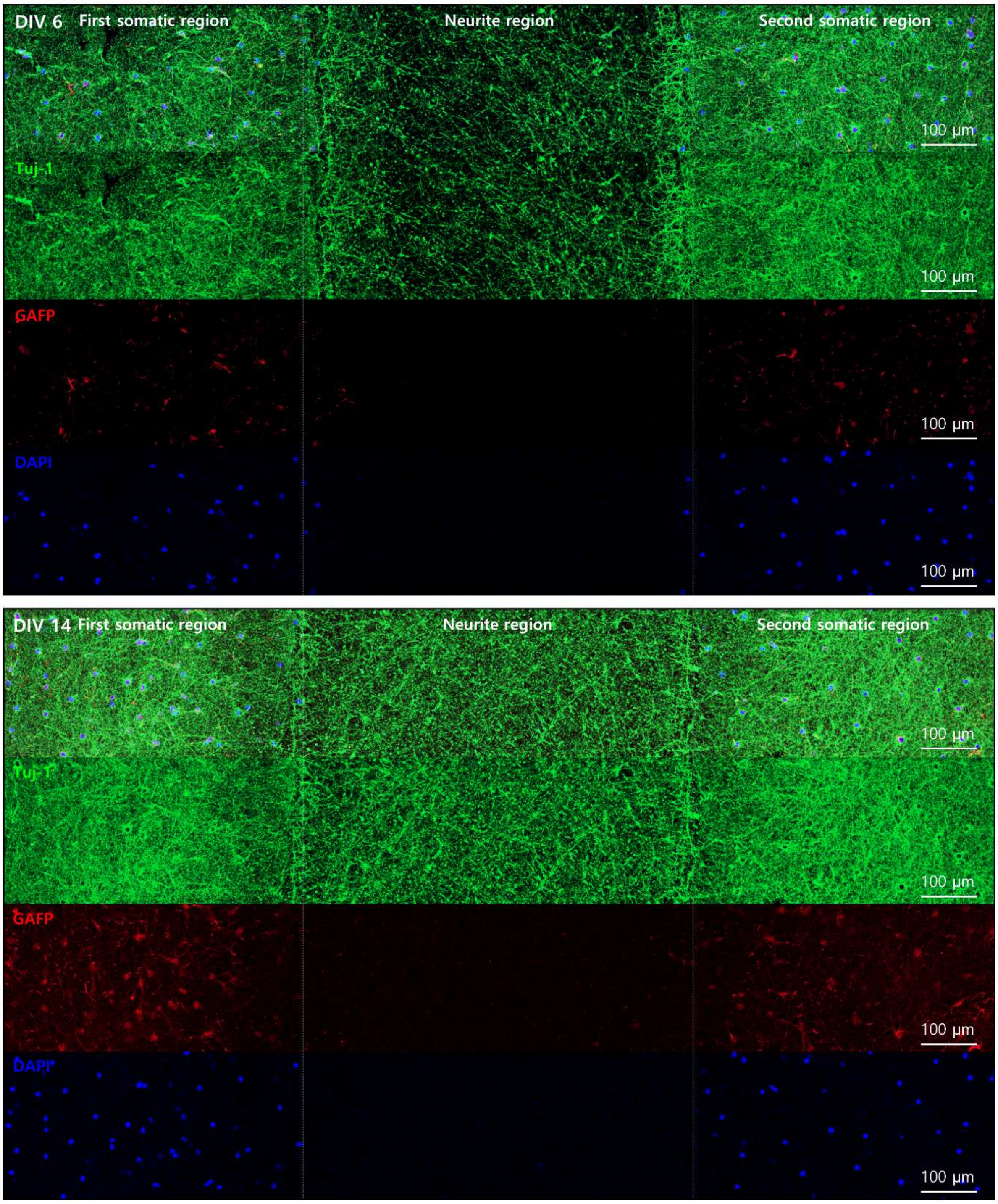

Supplementary Figure 12: The structural connection between two somatic regions at DIV 6 and DIV 14in the compartmentalized two-group 3D neural network model: Representative confocal fluorescence merged or respective images of immunostained neurites (Tuj-1, green-fluorescent), astrocyte (GFAP, red-fluorescent), and cell nucleus (DAPI, blue-fluorescent). Immunostaining and imaging were independently repeated at least three times with similar results to ensure reproducibility.

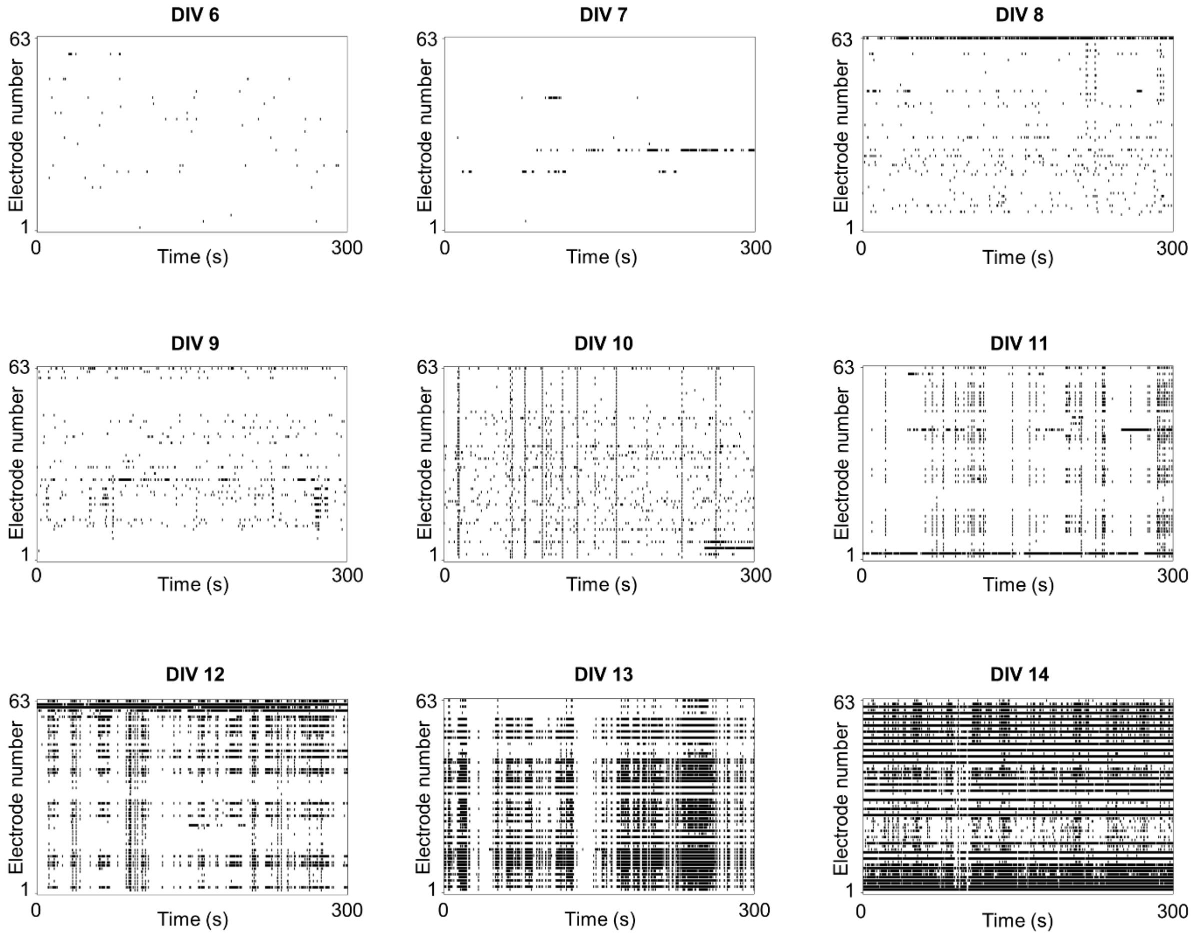

Supplementary Figure 13: Raster plots showing spontaneous activities recorded from 63 electrodes of the 3D multifunctional MEA from days in vitro (DIV) 6 to 14 in the single-group 3D neural network model.

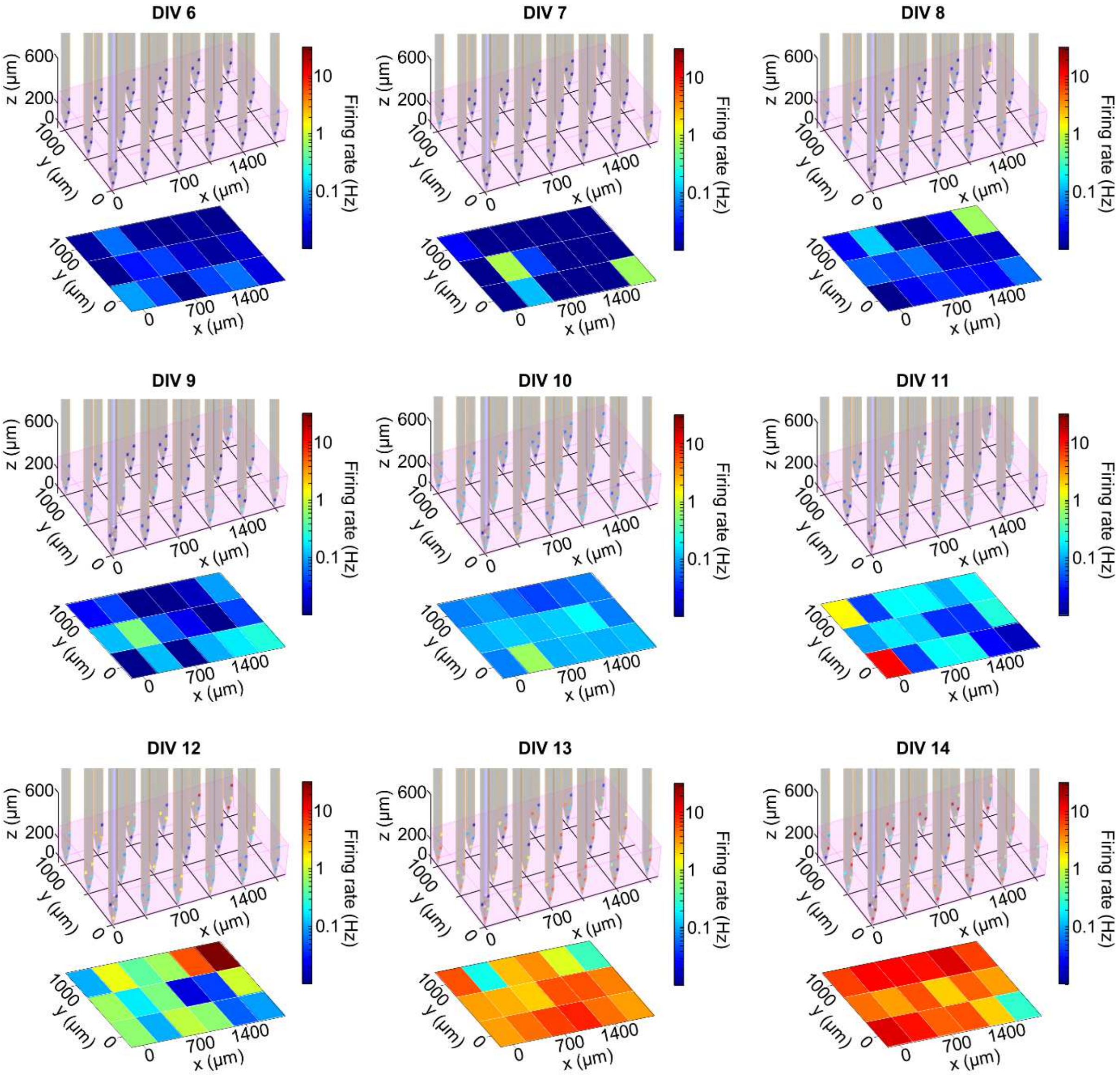

Supplementary Figure 14: 3D visualization of shank map in the single-group neural network model (top) and *z*-averaged map (bottom), colour-mapped firing rate from days in vitro (DIV) 6 to 14.

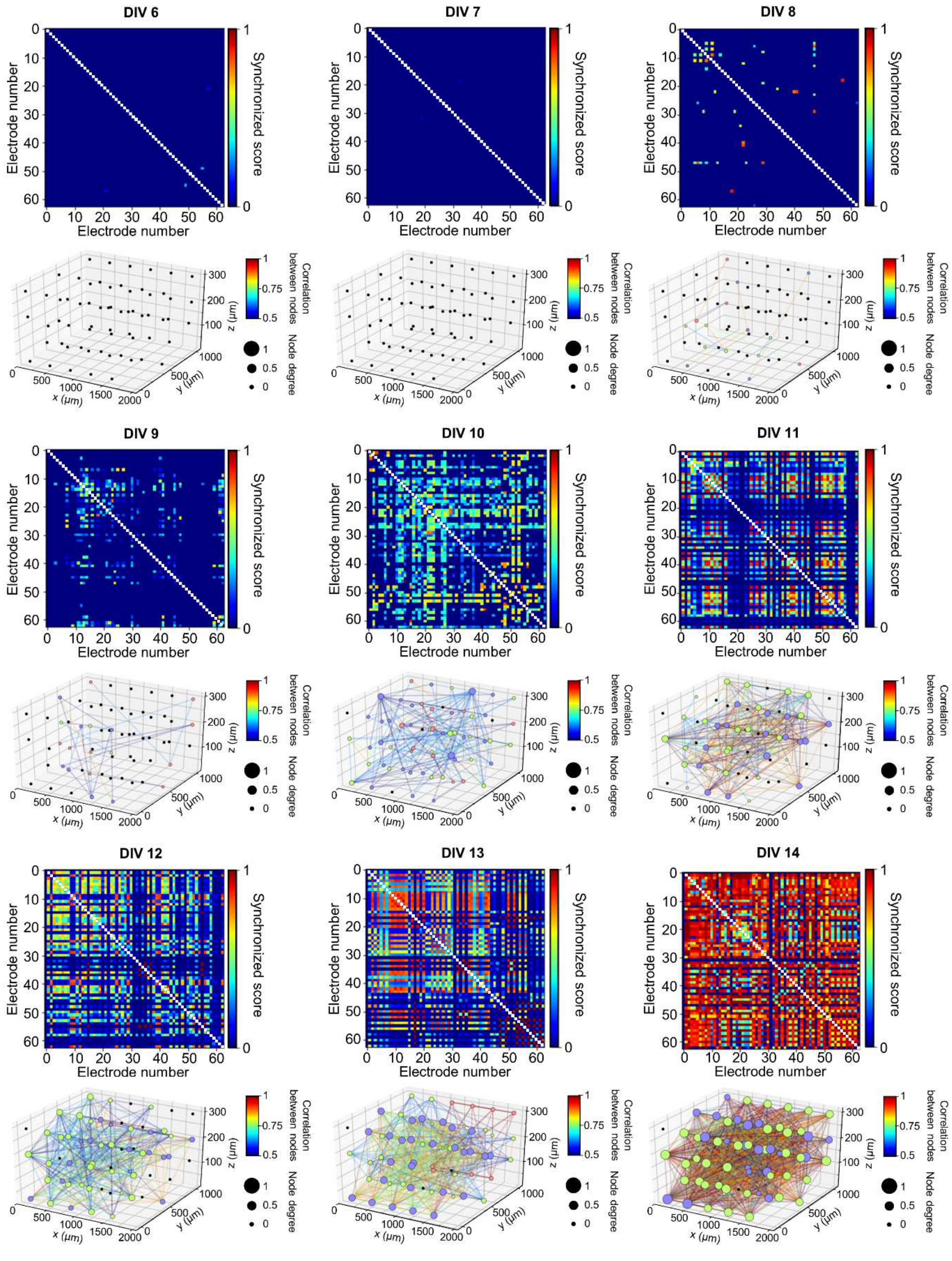

Supplementary Figure 15: Colour-mapped cross-correlation matrices displaying synchronized scores between electrodes and 3D network maps showing connectivities with node degrees as well as correlations between nodes, based on spontaneous activities from days in vitro (DIV) 6

to 14 in the single-group 3D neural network model. Node colour indicates network index connected among electrodes. Node degree indicates the number of connected electrodes from each electrode. Line colour indicates the correlation between electrodes.

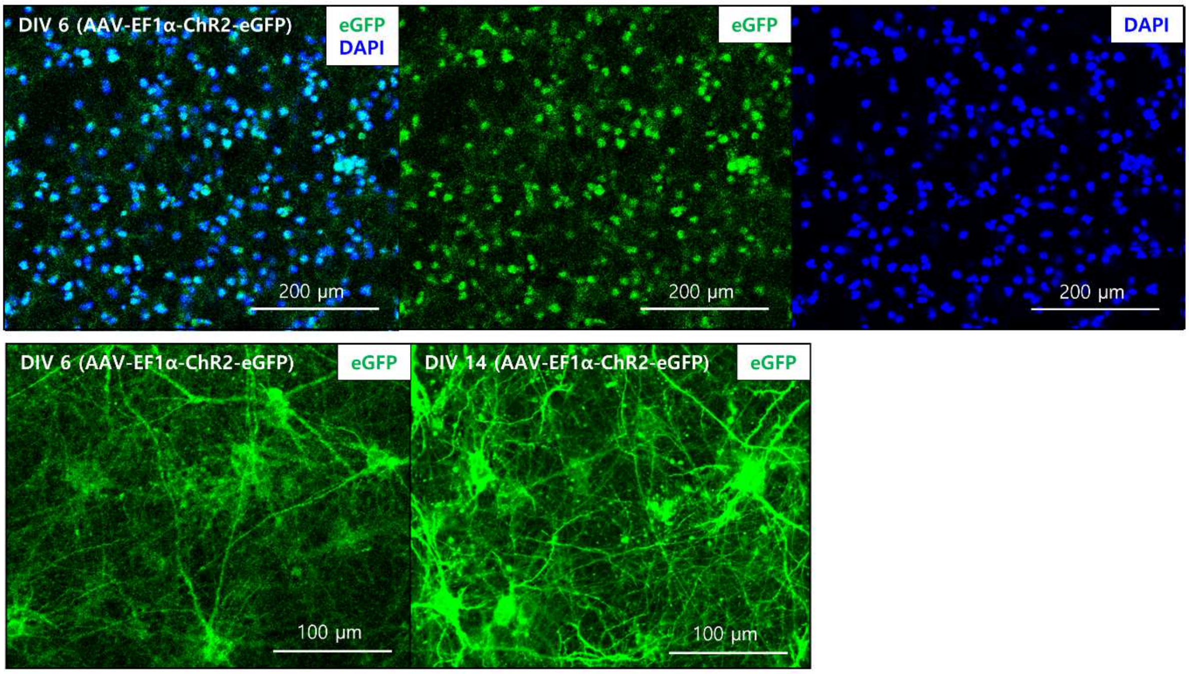

Supplementary Figure 16: Representative confocal fluorescence images of virus-infected neurons (eGFP) and cell nucleus (DAPI) at DIV 6 using 10x objective (top images) and confocal fluorescence images of virus-infected neurons (eGFP) at DIV 6 and DIV 14 using 20x objective (bottom images). DAPI staining and imaging were independently repeated at least three times with similar results to ensure reproducibility.

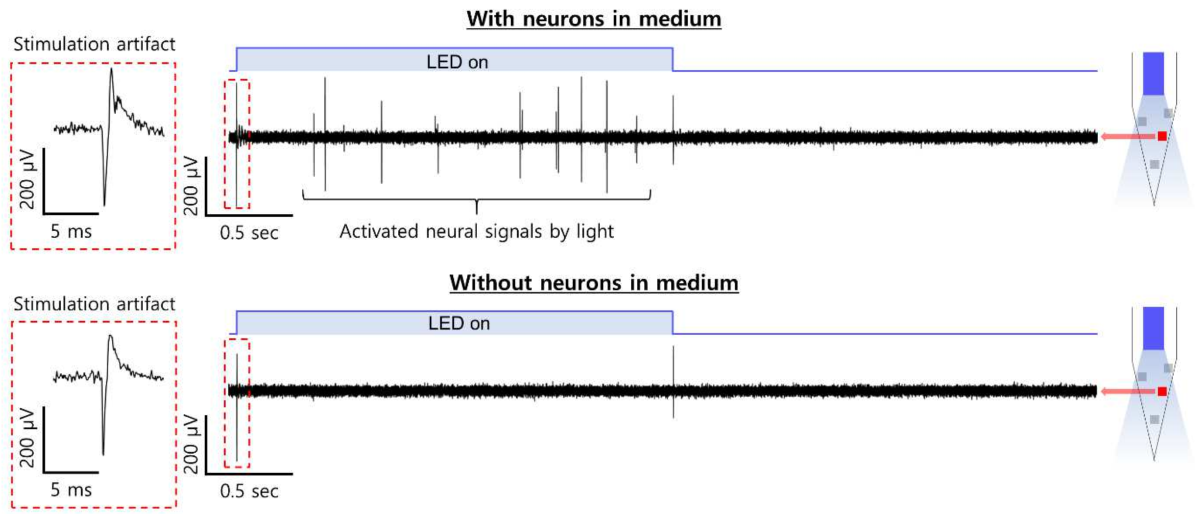

Supplementary Figure 17: Response by light stimulation with or without neurons in culture media and representative waveforms of stimulation artifacts recorded from the black Pt electrode on the multifunctional shank. The fibre tip's output optical power density was 76 mW·mm^{-2}, applied to all other in vitro experiments. Red-coloured electrodes indicates measured electrodes.

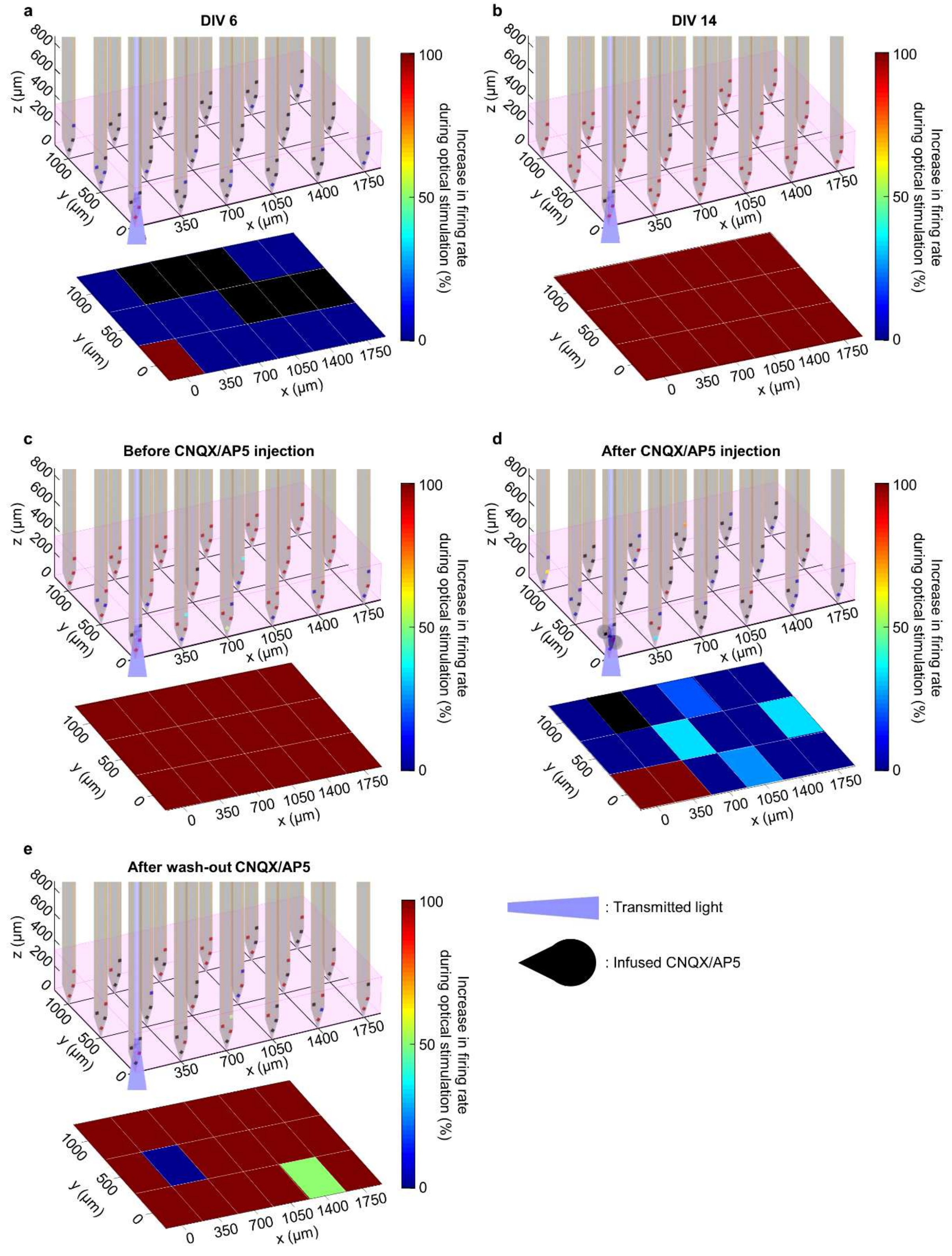

Supplementary Figure 18: 3D visualization of shank map in the single-group neural network model (top) and *z*-averaged map (bottom), colour-mapped increase in firing rate during LED on-cycles, compared with that during LED off-cycles at DIV 6 (a), DIV 14 (b), before (c) and after (d)

CNQX/AP5 injection, and after wash-out CNQX/AP5. Black-coloured circle indicates no signals recorded from electrodes. The light blue rectangle indicates the onset of light. Also, Black-coloured water drop indicates infused drugs.

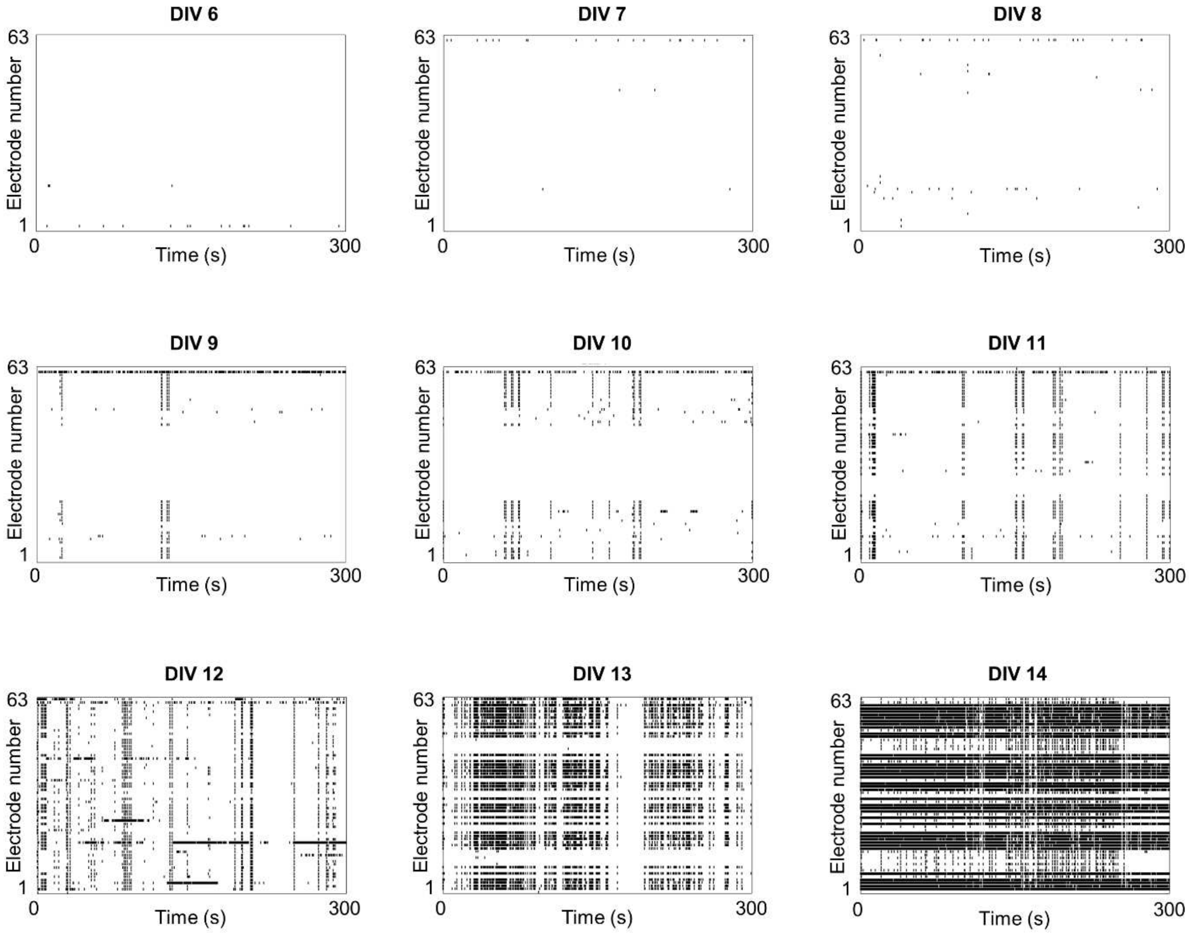

Supplementary Figure 19: Raster plots showing spontaneous activities recorded from 63 electrodes of the 3D multifunctional MEA from days in vitro (DIV) 6 to 14 in the compartmentalized two-group 3D neural network model.

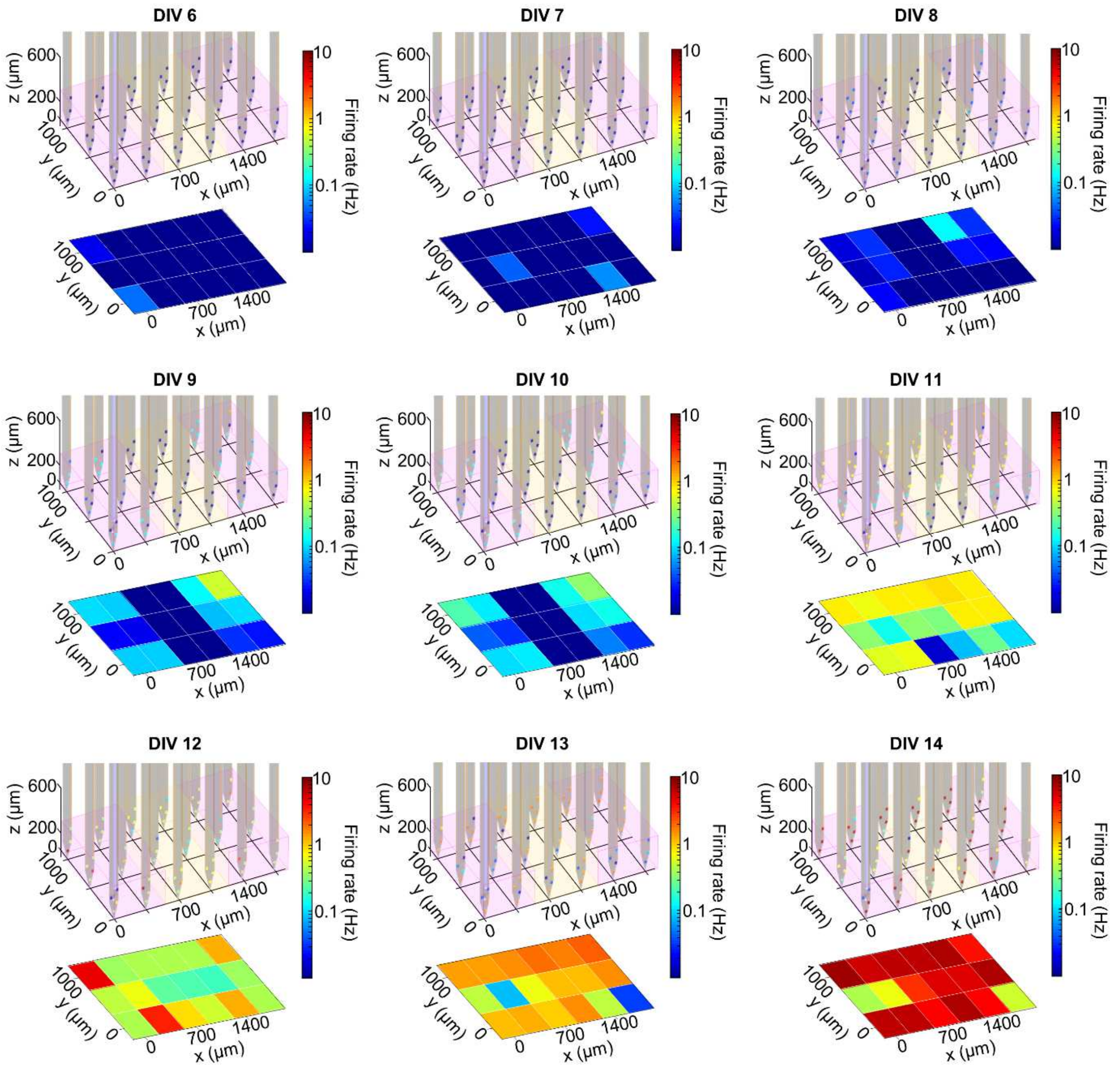

Supplementary Figure 20: 3D visualization of shank map in the compartmentalized two-group neural network model (top) and *z*-averaged map (bottom), colour-mapped firing rate from days in vitro (DIV) 6 to 14. Blue-coloured trapezoid indicates transmitted light from the LED.

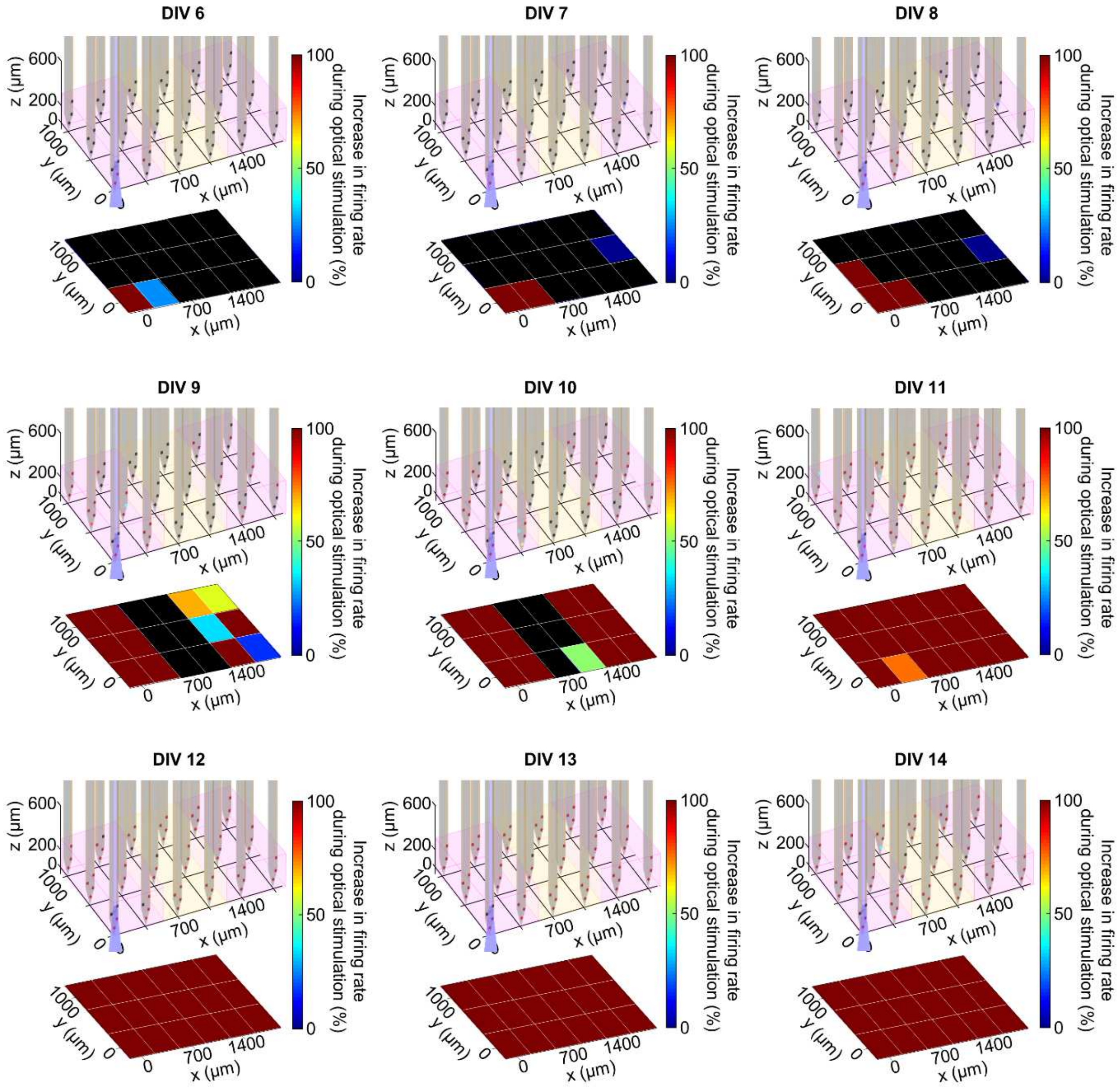

Supplementary Figure 21: 3D visualization of shank map in the compartmentalized two-group neural network model (top) and *z*-averaged map (bottom), colour-mapped increase in firing rate during LED on-cycles, compared with that during LED off-cycles from days in vitro (DIV) 6 to 14. Black-coloured circle indicates no signals recorded from electrodes. Also, The light blue rectangle indicates the onset of light.

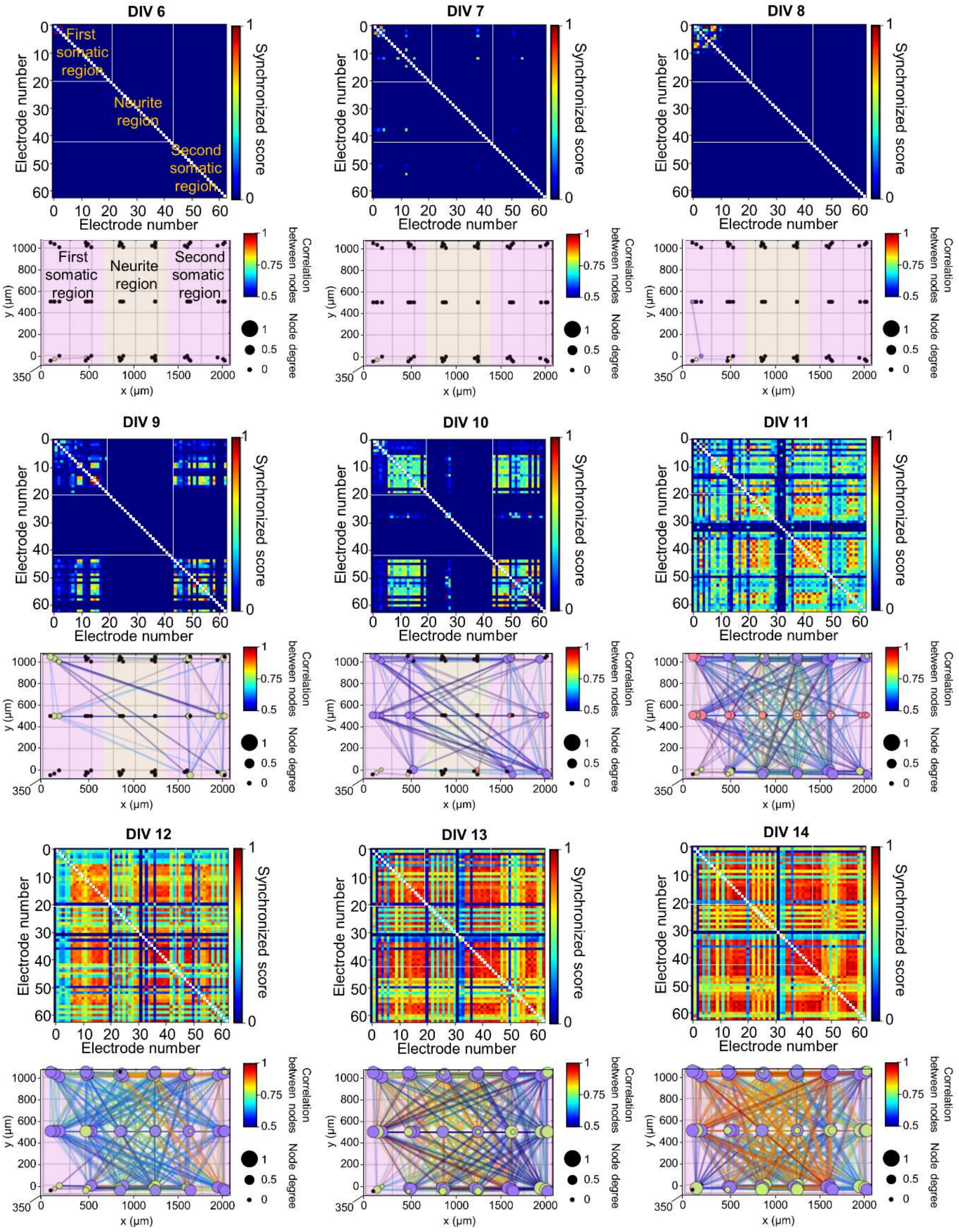

Supplementary Figure 22: Colour-mapped cross-correlation matrices displaying synchronized scores between electrodes and 3D network maps showing connectivities with node degrees as well as correlations between nodes, based on neural activities by optical stimulation from days in vitro (DIV) 6 to 14 in the compartmentalized two-group 3D neural network model. Node colour

indicates network index connected among electrodes. Node degree indicates the number of connected electrodes from each electrode. Line colour indicates the correlation between electrodes.

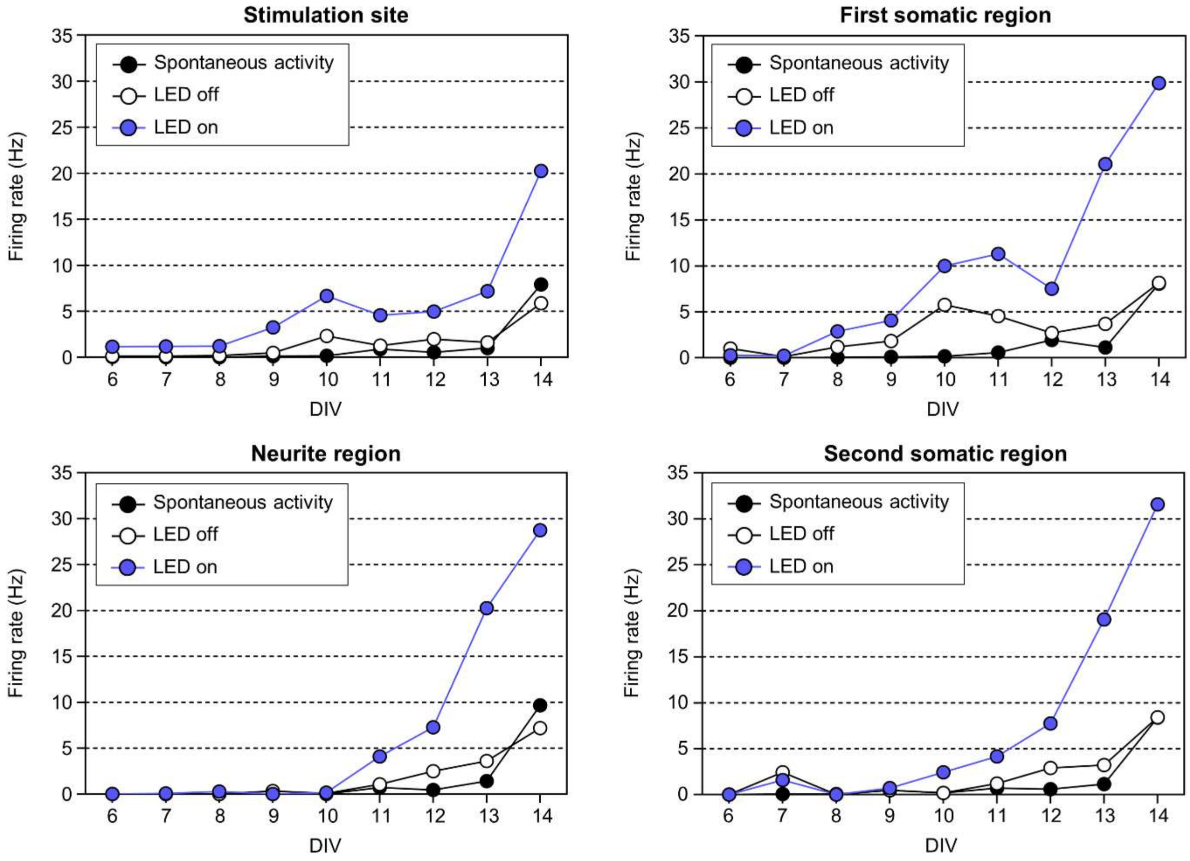

Supplementary Figure 23: Line graphs showing change of the firing rate of the spontaneous activity (black), during LED off (white)- and on (blue)-cycles of the optical stimulations at the stimulation site, in the first somatic, neurite, and the second somatic regions from DIV 6 to DIV 14 in the compartmentalized two-group 3D neural network model. Each circle indicates the firing rate at DIV.

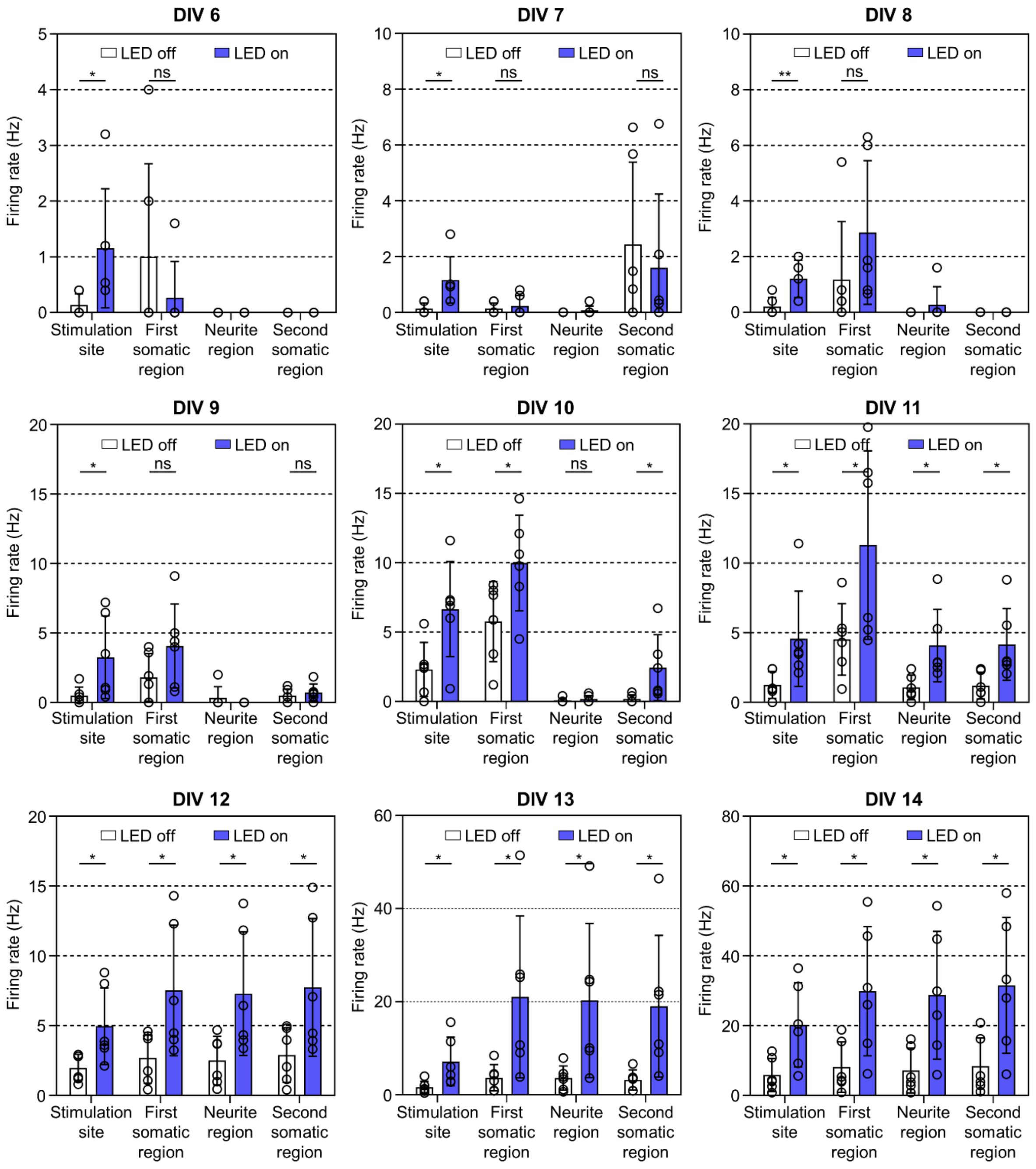

Supplementary Figure 24: Firing rate of 3D cultured neurons at the stimulation site, in the first somatic, neurite, and the second somatic regions during LED off (white)- and on (blue)-cycles of the optical stimulations at DIV 6. (*P = 0.0443 at the stimulation site; ns P = 0.3409 in the first somatic region), **DIV 7** (*P = 0.0143 at the stimulation site; ns P = 0.5737 in the first somatic region; ns P = 0.6148 in the second somatic region), **DIV 8** (**P = 0.0084 at the stimulation site; ns P = 0.2375 in the first somatic region), **DIV 9** (*P = 0.0494 at the stimulation site; ns P = 0.1424 in the first somatic region; ns P = 0.4864 in the second somatic region), **DIV 10** (*P = 0.0220 at the stimulation site; *P = 0.0444 in the first somatic region; ns P = 0.4506 in the neurite region; *P = 0.0436 in the second somatic region), **DIV 11** (*P = 0.0449 at the stimulation site; *P = 0.0449 in the first somatic region; *P = 0.0226

in the neurite region; *P = 0.0246 in the second somatic region), **DIV 12** (*P = 0.0303 at the stimulation site; *P = 0.0403 in the first somatic region; *P = 0.0337 in the neurite region; *P = 0.0495 in the second somatic region), **DIV 13** (*P = 0.0298 at the stimulation site; *P = 0.0364 in the first somatic region; *P = 0.0353 in the neurite region; *P = 0.0303 in the second somatic region), **DIV 14** (*P = 0.0218 at the stimulation site; *P = 0.0229 in the first somatic region; *P = 0.0214 in the neurite region; *P = 0.0228 in the second somatic region). Data are presented as mean values +/- s.d. with individual data points (white circle; n = 6 stimulation trials for all data). Statistical significance was tested with a two-tailed unpaired t-test.

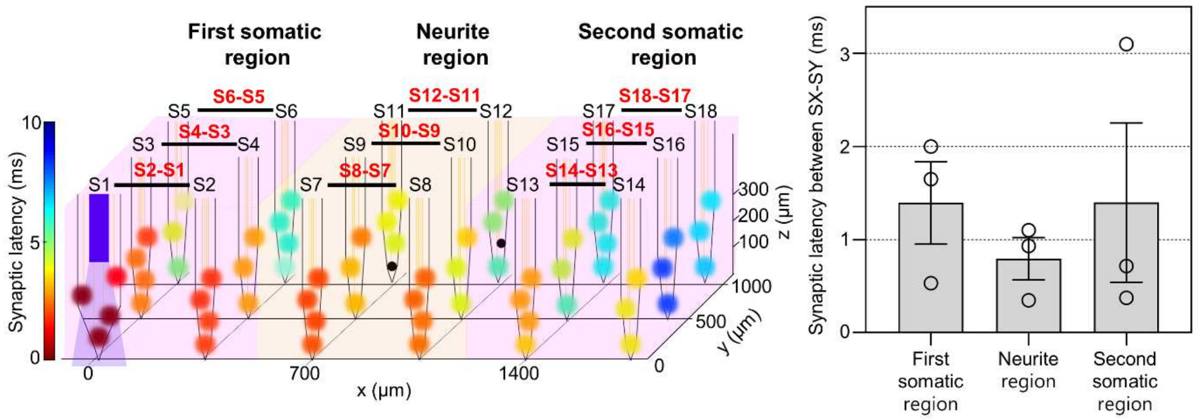

Supplementary Figure 25: Synaptic latency along the longitudinal direction in each region. 3D colour-mapped synaptic latency at each electrode, relative to the optical stimulation on shank 1. Black-coloured circle indicates no signals recorded from the electrodes. The light blue indicates transmitted light from the shank 1. “SX-SY” denotes a notation to estimate synaptic latency between SX and SY (bold red with underbar) along the longitudinal direction. (right) Bar graph showing the synaptic latency between SX-SY in somatic and neurite regions. Data are presented as mean values +/- s.d. with individual data points (white circle; *n* = 3 synaptic latency between SX-SY for each region (e.g., Synaptic latencies between S2-S1, S4-S3, and S6-S5 are included in first region)).

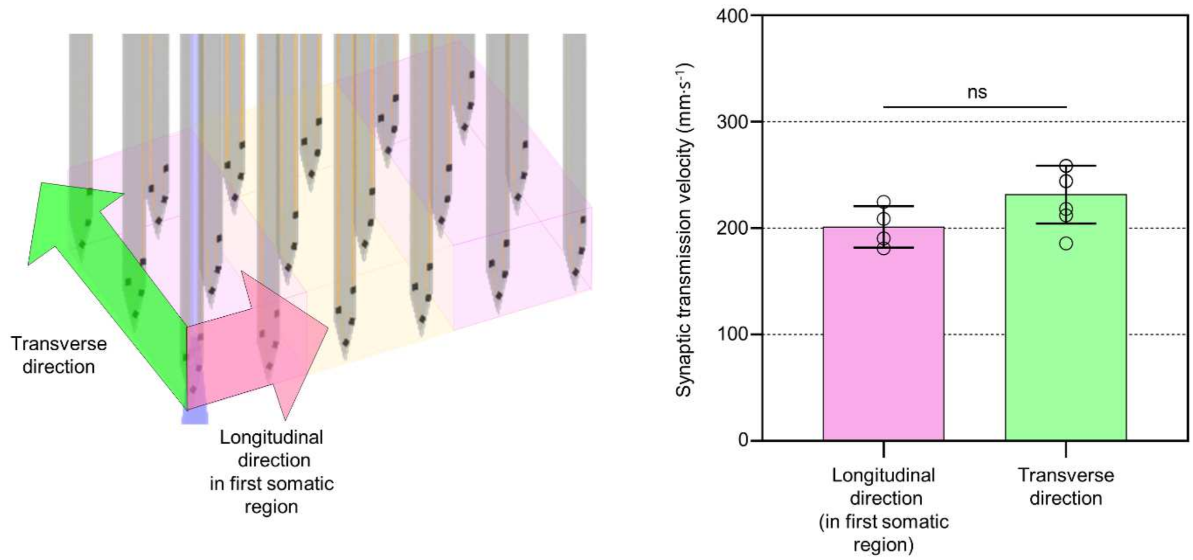

Supplementary Figure 26: Comparison of the synaptic transmission velocity along the transverse and longitudinal direction in the first somatic region. (left) Schematic illustration depicting the transverse (green) and longitudinal (pink) directions from the optical stimulation site. **(right)** Bar graph showing synaptic transmission velocity along the transverse and longitudinal directions in the first somatic region. Data are presented as mean values +/- s.d. (n = 7 the signal recorded electrodes along the transverse direction and n = 4 the signal recorded electrodes along the longitudinal direction). ns P = 0.0833. Statistical significance was tested with a two-tailed unpaired t-test.

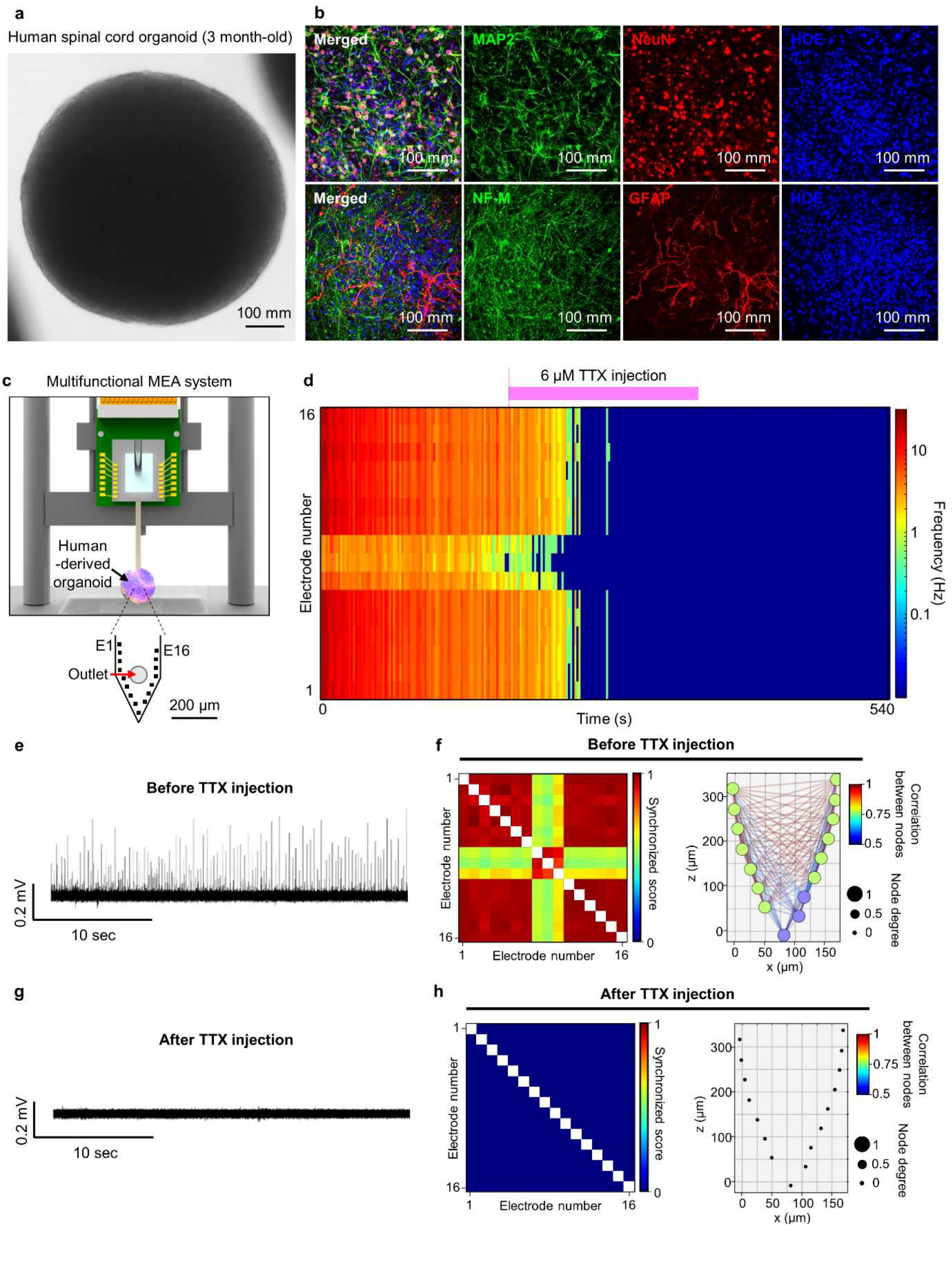

Supplementary Figure 27: Investigation and modulation of the connectivity in the human-derived spinal cord organoid. a Representative bright field image of human-derived spinal cord organoid. **b** Distribution of neuronal and non-neuronal population in the 3 month-old human-derived spinal cord organoid. Top images display double-immunofluorescent labeling of MAP2 (green) and

NeuN (red). Bottom images show double-immunofluorescent labeling of Neurofilament-M (green) and GFAP (red). Nuclei were counterstained with Hoechst (blue). **(a-b)** Immunostaining and imaging were independently repeated at least three times with similar results to ensure reproducibility. **c** Schematic diagram of functional analysis from human-derived organoid using a multifunctional neural probe system. **d** Colour-mapped raster plots showing neural activities recorded from 16 electrodes before and after 6 μM TTX injection. **e** Representative transient plot showing neural activities in human-derived spinal cord organoid before TTX injection. **f** Colour-mapped cross-correlation matrices displaying synchronized scores between electrodes and 2D network maps showing connectivities among electrodes before TTX injection. Node colour indicates network index connected among electrodes. Node degree indicates the number of connected electrodes from each electrode. Line colour indicates the correlation between electrodes. **g** Representative transient plot showing neural activities in human-derived spinal cord organoid after TTX injection. **h** Colour-mapped cross-correlation matrices displaying synchronized scores between electrodes and 2D network maps showing connectivities among electrodes after TTX injection. Node colour indicates network index connected among electrodes. Node degree indicates the number of connected electrodes from each electrode. Line colour indicates the correlation between electrodes.

Supplementary Note 1: Calculation of power efficiency from the LED to the fibre tip

To calculate light coupling efficiency from the LED to the fibre tip, we first calculated the LED's luminous efficiency. While the theoretical maximum luminous efficacy (K_{Max}) is 683 lm·W^{-1}, the luminous efficiency of the LED (K_{LED}) used in this study is 35.2 lm·W^{-1} based on datasheet. The luminous efficiency of radiation is expressed as a percentage[1]. The value of 683 lm·W^{-1} corresponds to an efficiency of 100%. Thus, the luminous efficiency of radiation (η) of the LED can be expressed as:

$$\eta = \frac{K_{LED}}{K_{Max}} \cdot 100\ (\%) \tag{1}$$

Based on Equation 1, the luminous efficiency of the LED is 5.15%. Thus, when the electrical power applied to the LED is 1 W, the optical power of emitted light is 51.5 mW. Next, when 51.5 mW of input optical power was applied from the LED, output optical power from the fibre tip was 150 µW. Then, we calculated light coupling efficiency based on the calculated input optical power (P_{in}) and the measured output optical power (P_{out}). The light coupling efficiency (η) can be expressed as:

$$\eta = \frac{P_{out}}{P_{in}} \cdot 100\ (\%) \tag{2}$$

Supplementary Reference

1. Choudhury, A.K.R., *et al. Principles of colour and appearance measurement: Object appearance, colour perception and instrumental measurement*. Elsevier, (2014).
