# Supplementary material for: 3D high-density microelectrode array with optical stimulation and drug delivery for investigating neural circuit dynamics
Source: Nat Commun. 2021 Jan 21;12:492. doi: 10.1038/s41467-020-20763-3 (PMC7820464; doi:10.1038/s41467-020-20763-3)
Supplement: Supplementary file 7 — Reporting Summary [file 41467_2020_20763_MOESM7_ESM.pdf]

nature research

Corresponding author(s): Il-Joo Cho

Last updated by author(s): Dec 7, 2020

Reporting Summary

Nature Research wishes to improve the reproducibility of the work that we publish. This form provides structure for consistency and transparency in reporting. For further information on Nature Research policies, see our Editorial Policies and the Editorial Policy Checklist.

Statistics

For all statistical analyses, confirm that the following items are present in the figure legend, table legend, main text, or Methods section.

n/a	Confirmed	
☐	☒	The exact sample size (*n*) for each experimental group/condition, given as a discrete number and unit of measurement
☐	☒	A statement on whether measurements were taken from distinct samples or whether the same sample was measured repeatedly
☐	☒	The statistical test(s) used AND whether they are one- or two-sided<br>*Only common tests should be described solely by name; describe more complex techniques in the Methods section.*
☒	☐	A description of all covariates tested
☒	☐	A description of any assumptions or corrections, such as tests of normality and adjustment for multiple comparisons
☐	☒	A full description of the statistical parameters including central tendency (e.g. means) or other basic estimates (e.g. regression coefficient) AND variation (e.g. standard deviation) or associated estimates of uncertainty (e.g. confidence intervals)
☐	☒	For null hypothesis testing, the test statistic (e.g. *F*, *t*, *r*) with confidence intervals, effect sizes, degrees of freedom and *P* value noted<br>*Give P values as exact values whenever suitable.*
☒	☐	For Bayesian analysis, information on the choice of priors and Markov chain Monte Carlo settings
☐	☒	For hierarchical and complex designs, identification of the appropriate level for tests and full reporting of outcomes
☒	☐	Estimates of effect sizes (e.g. Cohen's *d*, Pearson's *r*), indicating how they were calculated

Our web collection on statistics for biologists contains articles on many of the points above.

Software and code

Policy information about availability of computer code

Data collection	RHD200 USB interface software (Version 1.5) (Intan technologies), Matlab2016a (Matlab), ZEN microscope software 3.1 (Zeiss), NanoZ 1.4 (Neuralynx)
Data analysis	Matlab2016a (Matlab) was used to detect neural spikes.<br>Python 3.7.4 (Python software foundation) was used to analyze synchronized scores between electrodes and to visualize the network between electrodes. The open source packages Py spike and python-louvain, which are available at https://github.com/mariomulansky/PySpike/ and https://github.com/taynaud/python-louvain/. Also, custom code used for visualization of 3D network maps is freely available at https://github.com/Hyogeun-Shin/Visualization-of-3D-network-map/tree/v1.0.0 (https:// doi.org/10.5281/zenodo.4306072).<br>GraphPad Prism 7.04 (GraphPad Prism) was used to display the graphs and to perform statistical analysis.

For manuscripts utilizing custom algorithms or software that are central to the research but not yet described in published literature, software must be made available to editors and reviewers. We strongly encourage code deposition in a community repository (e.g. GitHub). See the Nature Research guidelines for submitting code & software for further information.

Data

Policy information about availability of data

All manuscripts must include a data availability statement. This statement should provide the following information, where applicable:

- Accession codes, unique identifiers, or web links for publicly available datasets
- A list of figures that have associated raw data
- A description of any restrictions on data availability

The authors declare that all data supporting the findings of this study are available within the article and its supplementary information files or from the corresponding author upon reasonable request. Source data are provided with this paper.

Field-specific reporting

Please select the one below that is the best fit for your research. If you are not sure, read the appropriate sections before making your selection.

- [x] Life sciences
- [ ] Behavioural & social sciences
- [ ] Ecological, evolutionary & environmental sciences

For a reference copy of the document with all sections, see nature.com/documents/nr-reporting-summary-flat.pdf

Life sciences study design

All studies must disclose on these points even when the disclosure is negative.

Sample size	Sample size is indicated in th figure legend for each experiment. We did not use a computational method to determine sample size. We chose based on previous experience with signal analysis from in vivo and in vitro models and determined sample size to be appropriate based on the consistency and magnitude of measurable differences in experiments.
Data exclusions	No data were excluded.
Replication	All experiments were repeated independently at least three times with similar result. All replicates were successfully performed.
Randomization	Samples were randomly assigned to experiments.
Blinding	The investigators were not blinded to group allocation during data collection. Blinding was not necessary because all data analysis was performed automatically using MATLAB and Python, with the same code run on each experiments.

Reporting for specific materials, systems and methods

We require information from authors about some types of materials, experimental systems and methods used in many studies. Here, indicate whether each material, system or method listed is relevant to your study. If you are not sure if a list item applies to your research, read the appropriate section before selecting a response.

Materials & experimental systems

n/a	Involved in the study	
☐	☒	Antibodies
☒	☐	Eukaryotic cell lines
☒	☐	Palaeontology and archaeology
☐	☒	Animals and other organisms
☒	☐	Human research participants
☒	☐	Clinical data
☒	☐	Dual use research of concern

Methods

n/a	Involved in the study	
☒	☐	ChIP-seq
☒	☐	Flow cytometry
☒	☐	MRI-based neuroimaging

Antibodies

Antibodies used	We used mouse anti-β-Tubulin III antibody (Sigma-Aldrich, T8578, 2G10, monoclonal, 1:200), chicken anti-GFAP antibody (Sigma-Aldrich, AB5541, polyclonal, 1:200), rabbit anti-NeuN antibody (Millipore, ABN78, polyclonal, 1:1000), chicken anti-MAP2 antibody (Millipore, AB5543, polyclonal, 1:5000), mouse anti-Neurofilament-M antibody (DSHB, 2H3, monoclonal, 1:250), rat anti-GFAP antibody (Invitrogen, 13-0300, 2.2B10, monoclonal, 1:500),<br>goat anti-mouse conjugated Alexa Fluor 488 antibody (Invitrogen, A-11001, polyclonal, 1:200), goat anti-chicken conjugated Alexa Fluor 647 antibody (Abcam, ab150171, polyclonal, 1:200), donkey anti-rabbit conjugated Cy3 antibody (Jackson, 711-165-152, polyclonal, 1:500), donkey anti-chicken conjugated Alexa Fluor 488 antibody (Jackson, 703-545-155, polyclonal, 1:500), donkey anti-mouse conjugated Alexa Fluor 488 antibody (Invitrogen, A21202, polyclonal, 1:500), donkey anti-rat conjugated Cy3 antibody (Jackson, 712-166-150, polyclonal, 1:500).
Validation	Antibody validation was confirmed the each manufacturer. Antibody validation was confirmed from each manufacturer. Mouse anti-β-Tubulin III antibody (Sigma-Aldrich, T8578, 2G10, monoclonal) was validated for species reactivity to human, rat, and mouse. Also, this antibody was suitable validated for applications in immunocytochemistry (ICC), immunohistochemistry (IHC), immunoprecipitation (IP), and western blot (WB). Chicken anti-GFAP antibody (Sigma-Aldrich, AB5541, polyclonal) was validated for species reactivity to human, bovine, pig, rat, and mouse. Also, this antibody was suitable validated for applications in ICC, IHC, and WB. Rabbit anti-NeuN antibody (Millipore, ABN78, polyclonal) was validated for species reactivity to mouse, snail, rat, and human. Also, this antibody was suitable validated for applications in ICC, immunofluorescence (IF), IHC, and WB. Chicken anti-MAP2 antibody (Millipore, AB5543, polyclonal) was validated for species reactivity to rat. Also, it is expected that the antibody will react with all mammals. This antibody was suitable validated for applications in ICC, IHC, and WB. Mouse anti-Neurofilament-M antibody (DSHB, 2H3, monoclonal) was validated for species reactivity to human, rat, and mouse. Also, this antibody was suitable validated for applications in IHC, IF, and WB. Rat anti-GFAP antibody (Invitrogen, 13-0300, 2.2B10, monoclonal) was validated for species reactivity to human, rat, mouse, bovine, and guinea pig. Also, this antibody was suitable validated for applications in ICC, IHC, IP, and WB.

Animals and other organisms

Policy information about studies involving animals; ARRIVE guidelines recommended for reporting animal research

Laboratory animals	Pregnant Sprague Dawley (SD) rats (embryo 18) were used in the study.
Wild animals	No wild animals were used in the study.
Field-collected samples	No field collected samples were used in the study.
Ethics oversight	All procedures except human-derived pluripotent stem cell (PSC) related experiment were conducted according to the animal welfare guidelines approved by the Institutional Animal Care and Use Committee of the Korea Institute of Science and Technology. The human PSC related experiment was approved by the Korea University Institutional Review Board.

Note that full information on the approval of the study protocol must also be provided in the manuscript.
